# Mixed-Forest Species Establishment in a Monodominant Forest in Central Africa: Implications for Tropical Forest Invasibility

**DOI:** 10.1371/journal.pone.0097585

**Published:** 2014-05-20

**Authors:** Kelvin S.-H. Peh, Bonaventure Sonké, Olivier Séné, Marie-Noël K. Djuikouo, Charlemagne K. Nguembou, Hermann Taedoumg, Serge K. Begne, Simon L. Lewis

**Affiliations:** 1 Institute for Life Sciences, University of Southampton, Southampton, United Kingdom; 2 Ecology and Global Change group, School of Geography, University of Leeds, Leeds, United Kingdom; 3 Conservation Science Group, Department of Zoology, University of Cambridge, Cambridge, United Kingdom; 4 Plant Systematic and Ecology Laboratory, Higher Teacher’s Training College, University of Yaoundé I, Yaoundé, Cameroon; 5 Institute of Agricultural Research for Development, Yaoundé, Cameroon; 6 Department of Botany and Plant Physiology. University of Buea, Buea, Cameroon; 7 African Forest Forum, Nairobi, Kenya; 8 Cameroon Biodiversity Conservation Society, Yaoundé, Cameroon; 9 Department of Geography, University College London, London, United Kingdom; Cirad, France

## Abstract

**Background:**

Traits of non-dominant mixed-forest tree species and their synergies for successful co-occurrence in monodominant *Gilbertiodendron dewevrei* forest have not yet been investigated. Here we compared the tree species diversity of the monodominant forest with its adjacent mixed forest and then determined which fitness proxies and life history traits of the mixed-forest tree species were most associated with successful co-existence in the monodominant forest.

**Methodology/Principal Findings:**

We sampled all trees (diameter in breast height [dbh]≥10 cm) within 6×1 ha topographically homogenous areas of intact central African forest in SE Cameroon, three independent patches of *G. dewevrei*-dominated forest and three adjacent areas (450–800 m apart). Monodominant *G. dewevrei* forest had lower sample-controlled species richness, species density and population density than its adjacent mixed forest in terms of stems with dbh≥10 cm. Analysis of a suite of population-level characteristics, such as relative abundance and geographical distribution, and traits such as wood density, height, diameter at breast height, fruit/seed dispersal mechanism and light requirement–revealed after controlling for phylogeny, species that co-occur with *G. dewevrei* tend to have higher abundance in adjacent mixed forest, higher wood density and a lower light requirement.

**Conclusions/Significance:**

Our results suggest that certain traits (wood density and light requirement) and population-level characteristics (relative abundance) may increase the invasibility of a tree species into a tropical closed-canopy system. Such knowledge may assist in the pre-emptive identification of invasive tree species.

## Introduction

Lowland rain forests are commonly diverse and complex. However, not all lowland forest communities show a particularly high tree alpha (α-) diversity and these low-diversity forests typify some forested areas in Malesia and the Neotropics, but are most common in Central Africa [1]–[6]. *Gilbertiodendron dewevrei* is a species which extensively dominates large patches of forests on the plateau in central Africa. These classical monodominant forests exist alongside higher-diversity forests often with sharp boundaries [3], [6].

*Gilbertiodendron dewevrei*-dominated forest often involves a large number of *G. dewevrei* co-existing with a number of other species occurring with low abundance and this might be expected to reduce the α-diversity in that area. For example, mixed forest is significantly more diverse than *G. dewevrei* forest based on trees with diameter in breast height (dbh)≥10 cm within 25 m×25 m plots [3]. However, Connell and Lowman (1989) have shown that although the canopy tree diversity negatively correlates with dominance by one canopy species within their study plots, increasing dominance has limited influence on the diversity of subcanopy trees [1]. Similarly, the number of species within the *G. dewevrei* forest over large areas is similar as compared to their adjacent mixed forests at the Ituri Forest of the Democratic Republic of the Congo; species richness of trees ≥10 cm dbh in 2×10 ha study plots in *G. dewevrei* forest (mean species number [±SD] based on the 1-ha scale = 56±24; range = 18–101 species per ha) was comparable to that from 2×10 ha plots in adjacent mixed forest (mean species number [±SD] based on the 1-ha scale = 68±8; range = 56–85 species per ha) [7]. By contrast, in 5×1 ha plots each of mixed and monodominant plots in Cameroon showed significantly higher species richness in the mixed forest than the monodominant forest (Shannon diversity index of tree species ≥10 cm: mixed forest = 5.70±0.28; monodominant forest = 2.29±0.48) [8]. However, each of these studies confounds differing stem numbers within the two forests, with a recent study showing a mean stem density (> = 10 cm dbh) of 434 ha^−1^ in mixed forest and 340 in *G. dewevrei* forest (n = 23) [9]. Therefore, it is unclear whether or not the high diversity in the *G. dewevrei* forests is due to tree species of the mixed forests not being excluded, but being present at reduced density [4].

Here we ask: is the species richness of the successfully established mixed-forest trees in the *G. dewevrei* forest (hereafter the monodominant forest) the same as its adjacent high-diversity forest (hereafter the mixed forest)? We use non-parametric species richness estimators–for the first time–to estimate the species richness of the *G. dewevrei* forest. We use as our study site the Dja Faunal reserve in south-eastern Cameroon.

We then address the question of whether successful establishment of the mix-forest trees in the monodominant forest is non-random. We compared the life-history attributes of species that were successfully established in the monodominant forest with those that were present in the adjacent mixed forest but were absent in the monodominant forest (i.e., unsuccessful). If fitness proxies and life-history traits have an impact on the recruitment of non-dominant mixed-forest species to maturity, we can then (1) conclude that the successful establishment of these species is non-random and (2) identify which subset of the pool of available mixed-forest species could potentially be successful in monodominant forests.

Monodominance in the *G. dewevrei* forests has been attributed to the life-history traits of dominant species such as ectomycorrhizal association, being shade tolerant, possessing closed canopy, producing large seeds, and creating deep leaf litter that slowly decomposed [3], [5], [6], [10]. It is proposed that the monodominance is due to several factors interacting in a positive feedback encouraging further establishment of *G. dewevrei*, and providing barriers to the establishment of non-dominant species [6], often in the absence of exogenous disturbance events [1]. It is not thought that differing edaphic factors are the cause of *G. dewevrei* monodominance [11]. Hence, this study demonstrates the utility of comparative studies based on species traits to show if the non-dominant mix-forest tree species are able to break through any barriers created by the dominant species and then establish to maturity. While it is possible that some individuals of non-dominant species may be persisting after ‘invasion’ by *G. dewevrei*, this is less likely when not near the edges of monodominant patches of forest; where the dominant species dominates all size classes of trees and understory seedlings. We investigate the likelihood of these possibilities as part of the study. Our study should provide fundamental knowledge for understanding the regeneration processes of the non-dominant species in the monodominant forests and perhaps assist in elucidating mechanisms that result in monodominance in these forests.

Our final objective in this study is to investigate the relative invasibility of tropical forest. In general, the life-history traits of the non-dominant mixed-forest tree species that are important for the successful establishment in monodominant forest may also be the attributes of an exotic tree species for successful invasion in a closed-canopy system. The knowledge of the traits that most associated with tropical forest invasions could be a step towards tangible conservation and identification of potential invasive species [12].

## Materials and Methods

### Study Area

**Figure 1:**
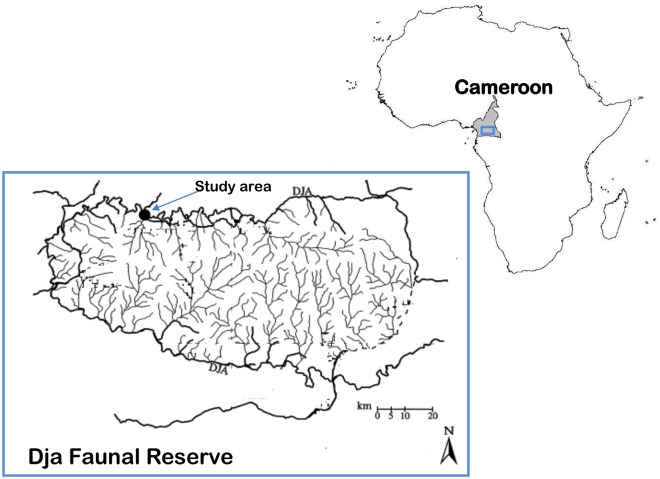
Map of study location at the Dja Faunal Reserve in Cameroon.

Our study was conducted at the Dja Faunal Reserve (hereafter called the reserve), located between 2°49′–3°23′N and 12°25′–13°35′E in south-eastern Cameroon (Fig. 1). Cameroon Ministry of Forests and Fauna (MINFOF) granted the permission for this work in this reserve. Established in 1950, the reserve covers an area of 526,000 ha, which consists of lowland moist evergreen tropical forests at an elevation between 400–800 m [13]. About two-third of the reserve’s perimeter is demarcated by the Dja River, forming a natural boundary. Such inaccessibility due to the natural barrier offers the reserve protection from large-scale human disturbance. Sampled forest stands are located in the north-western part of the reserve south of the village Somalomo. The region has an equatorial climate with the maximum average monthly temperature of 25.8°C in February, and minimum average monthly temperature of 23.6°C in October. The average annual rainfall of the area was 1512 mm. The climate is characterized by two wet seasons with rainfall peaks in May and October. The two dry periods are July–August and December–February [11].

The vegetation in the reserve has a main canopy of 30–40 m with tree emergents rising to 60 m [13]. Large naturally-occurring monodominant patches of *G. dewevrei* occur within the mixed forest throughout the reserve, although the total area of these monodominant forest patches in the reserve is not known. The ten most common tree species in the mixed forests in terms of number of individuals per ha are *Petersianthus macrocarpus*, *Carapa procera*, *Santira trimera*, *Polyalthia suaveolens*, *Heisteria trillesiana*, *Penthaclethra macrophylla*, *Anonidium mannii*, *Centroplacus glaucinus*, *Plagiostyles africana* and *Tabernaemontana crassa* [8]. For detailed description of the structure and flora composition of the mixed forests, see Sonké 2004 [14].

Soils of the region are often described as clayey and poor in nutrients [13]. Soils of the monodominant and mixed forests in the reserve are both acidic weathered clayey Ferrosols [11]. Physical and chemical soil properties were not significantly different between the two forest types (for description of soil analyses, see Peh *et al.* 2011 [11]). This conforms to findings in other studies [2], [3], [15]. Hence, the potential differences in diversity were unlikely to be due to edaphic conditions.

### Tree Sampling

The tree α-diversity in three monodominant forests and their adjacent mixed forests were sampled using 100 m×100 m (1 ha) plot surveys in February–March 2005 following the standardized guidelines [16], [17]. The three plots in monodominant forest were non-contiguous patches chosen based on satellite images and local knowledge. The location of the *Gilbertiodendron dewevrei*-dominated patches was at least 4 km apart from each other. For each *Gilbertiodendron dewevrei*-dominated plot, a corresponding plot in the adjacent mixed-species forest was established for comparative purposes. The three mixed forest plots were ranged between 452 m and 818 m away from their *Gilbertiodendron dewevrei*-dominated counterparts. Each plot was divided into 20 m×20 m quadrats where species were identified, as far as possible, for all tree stems with dbh≥10 cm. Tree was defined as free-standing woody stems. All scientific names for tree taxonomy in this study were standardized for orthography and synonyms with the African Flowering Plant database (http://www.ville-ge.ch/cjb/bd/africa/index.php). For unknown species, we collected voucher specimen for identification at the National Herbarium of Cameroon. We considered species in the monodominant forest to be locally common if there were three or more individuals found within our sampling area of 3 ha, following Hubbell and Foster (1986) [18]. To compare the patterns of stem size distribution between the monodominant and mixed forests, we considered for each forest type, the number of stems in different dbh classes (10–20 cm; >20–30 cm; >30–40 cm; >40–50 cm; >50–60 cm; and >60 cm) and the proportion of stems in different dbh classes. Finally, in March 2013, the plots were recensused, including newly recruited stems. This allows us to see if the monodominant forests are gaining or losing species over the eight-year period.

### Tree Species Traits and Fitness Proxies

We restricted our analyses to the 193 positively identified tree species from all study plots. To assess which species traits and fitness proxies correlate with successful establishment in the monodominant forests, we first compiled a checklist of all species found in the six hectares of forest sampled. We then compared the mixed forest checklist with that of the monodominant forest to identify the list of species that occurred in the plots of both forest types (n = 58 species).

We compiled ecological trait data that (a) are considered to confer competitive advantages [5], [7], and (b) could be obtained for at least 75% of the 193 species. Our analyses focused on the following ecological traits: (1) wood mass density; (2) plant height at maturity; (3) maximum dbh; (4) primary fruit/seed dispersal mechanism; and (5) ecological guild in terms of light requirement for seedling establishment. In addition to ecological traits, we also compiled the relative abundance and species geographical distribution data – both are proxies of the fitness of the species for the environment – and positively related to invasion success in other studies. Some traits such as ectomycorrhizal status and seed mass, which some evidence suggests are important for the establishment of the dominance of *G. dewevrei*, are missing, as we lack sufficient data. There is no database for the ectomycorrhizal status for African tree species so far, and the Kew Royal Botanic Gardens’ seed information database (http://data.kew.org/sid/sidsearch.html) does not have information on seed weight for many of the African species in this study.

The relative abundance of each species in the mixed forests was based on the three mixed forest plots. We obtained wood mass density, defined as dry wood mass/fresh wood volume (g.cm^−3^) [19], for each species that occurred in our plots from a compilation of 34 source in Lewis and others (2009) [20], and a wood density database (http://worldagroforestry.org/sea/products/AFDbases/WD/index.htm), with those reported as calculated at 12% moisture content were corrected using a calibration equation: ρ = 0.0134+0.800x, where x  =  wood mass density at 12% [21], [22]. From a total of 193 species, 79.3% have corresponding specific wood mass density values at the species level (153 cases). Wood density is a relatively conserved trait, and therefore closely related to phylogeny [22]. Therefore, for individual stems with no species-specific data, we took mean genus-level of wood density values (34 cases; 17.6% of 193 species). We classified 6 species (3.1%) as cases with undetermined value as these have no data even at the mean family-level.

We collected data on other traits for each species based on published literature [14], [23], herbarium specimens, and personal observations. We classified species categorically according to maximum stature in three classes based on the definition in Swaine and Whitmore (1988) [24]: large trees (>30 m tall), medium trees (10–30 m tall) and small trees (<10 m tall). We placed species on the basis of maximum dbh in three classes following Sonké (2004) [14]: large diameter (>100 cm in dbh), medium diameter (50 cm–100 cm) and small diameter (<50 cm). The maximum dbh of each species was based on the observations made in Sonké (2004) [14]. We ranked each species in one of two categories according to its fruit/seed dispersal mode (biotic and non-biotic dependent). Each species was classified into one of two categories according to its ecological guild in terms of light requirement [24], and each was grouped according to its geographical distribution (narrow: species endemic to lower-Guinea-Congolean biogeographical region; and wide: that includes species which were not endemic to the region [14]).

### Species Richness Estimations

Using subsamples of the 1-ha plots, we graphed sample-based rarefaction curves (i.e., equivalent to smoothed accumulation curves) rescaled to (i) the number of individuals to compare the number of tree species between the different forest types, and (ii) the number of samples (i.e., quadrats) to compare the density of forest species between these forest types. We then plotted curves of the number of individuals against the number of samples to compare the population density. Further, for statistical corroboration of potential differences in species richness between the monodominant and mixed forest, we compared the mean number of tree species per quadrat between forest types using the program EstimateS [25]. Thus, each forest type we utilised 75 quadrats of 20 m×20 m in total from the three plots, and the order of sampling was randomized 100 times for the rarefaction process [26]. The mean number of individuals observed per quadrat (i.e., stem density) for the monodominant forest was 13.68±0.82 (±95% CI; *n* = 3) and that for the mixed forest was 17.47±0.92 (±95% CI; *n* = 3).

Because rarefaction cannot be used for extrapolation from smaller samples, it does not provide an estimate of asymptotic species richness [27]. To estimate the tree species richness for each forest type, we generated non-parametric species richness estimators from EstimateS [25] based on the distribution of rare species in the community assemblage of each forest type [28]. We used nine different nonparametric species estimators: ACE (abundance-based coverage estimator), ICE (incidence-based coverage estimator), Chao1, Chao2, Jackknife1, Jackknife2, Michaelis-Menten, and bootstrap methods, because different estimators perform best for different data sets [29]. Further, we calculated the Fisher’s α value of each monodominant forest plot and the adjacent mixed forest plot as this is a commonly-reported metric in the ecological literature.

In addition, we used Entropart in the R package [30] – which employs the state-of-the-art method of entropy partitioning (see http://CRAN.R-project.org/package=entropart) – to estimate the effective species number (Hill number) of each forest type. This approach which assumes that community species follow multinomial distributions [31], [32] enables us to correct sampling biases and compare 95% confidence intervals between the two forest types.

### Statistical Analyses of Co-occurrence

To examine the effects of individual species traits on the recruitment to maturity in the monodominant forest, we performed two-variable binomial logistic regressions (logit model) between each trait and the status of each non-dominant tree species in the monodominant forest, categorized as presence (successful) or absent (unsuccessful). Our data precluded the use of the independent-contrasts approach [33] for controlling the effects of phylogenetic autocorrelations because of the inclusion of categorical variables (e.g., primary fruit/seed dispersal mechanism) in our analyses and the lack of complete phylogeny of the study taxa (O. Hardy, *pers. comm.*). As an alternative, we included family as a covariate in our statistical analyses. For families consist of less than 10 species in our sample, we lumped those individuals as belonging to a single family to prevent problem of low ‘cell count’ that may result in the regression not reaching convergence for parameter estimate [34]. Any phylogenetic effect at the genus level is likely to be weak because most genera in our data set are species poor with only 1.54 species per genus on average and 70% of species do not have congenerics. To assess the influence of phylogeny on the analyses, we repeated the same tests with each species trait as the sole independent variable without family as a covariate. These analyses allow unequal sample sizes for all variables (i.e., missing data). Nevertheless, the sample size for each variable was large (n≥144 species; at least 75% of the 193 species complete for each variable).

To determine the most parsimonious model that predicts successful establishment, we used Akaike’s Information Criteria (AIC) to find the model that has the best combination of significant life-history variables retained by the univariate analyses. However, we did not test all the possible permutations of all significant variables in order to keep the number of candidate models to the minimum [35]. Models that comprised only a single variable were excluded in the analysis because the establishment success is unlikely to be accounted for by only one variable. Since our sample size was relatively small, we employed the second-order model selection criterion AIC_c_, which is a small sample bias-corrected version of AIC [36]. For a model M_i_, AIC_c_ is expressed as: AIC_c_ = −2×log(likelihood)+2*K*+(2*K*[*K*+1])/(n−*K*−1), where log(likelihood) is the log-transformed value of the likelihood, *K* is the number of parameters, and n is the sample size [36].

If the global model (i.e., one that includes all variables retained by the univariate analyses) adequately described the data, then AIC_c_ selects a parsimonious model that fits [36]. To determine whether the global model had a good fit, we performed the Hosmer-Lemeshow goodness-of-fit test on the global model and the test statistics showed that our global model adequately fitted the data (χ^2^ = 9.38, d.f. = 8, *P*>0.05).

The best candidate model was identified by its lowest AIC_c_ difference and highest Akaike weight [36] and its variables were used to construct a multiple logistic regression model for predicting the successful establishment of a tree species in the monodominant forest. As the regression analysis is based on the assumption that the predictor variables are linearly independent of each other, we used the non-metric multidimensional scaling (NMDS) to check for multicollinearity among the variables [37]. NMDS is an ordination technique that is suitable for our data because it does not assume linear relationships and it allows the use of non-Euclidean (Bray-Curtis) distance measures for our nominal variables. The goal of using NMDS is to plot similar variables together and dissimilar ones further part in order to detect multicollinearity.

## Results

### Comparing Monodominant and Mixed Forest Diversity

In the 6 ha, we recorded a total of 2336 individual stems representing 211 identified tree species and morphotypes (194 species, 17 morphotypes; see Table S1 in Supporting Information). In the monodominant forest plots of 3 ha, there were 71 tree species (including *G. dewevrei* and 2 morphotypes) recorded. Ten tree species in the monodominant plots were not recorded in the mixed forest plots (see Table S1), although they do occur in mixed forest outside our plots [14]. Only two stems in the monodominant forests were treated as morphotypes. In the mixed forest plots of 3 ha, 198 tree species (including 15 morphotypes from 15 stems) were recorded. *G. dewevrei* was absent in the mixed forest. There were 125 tree species (excluding morphotypes) unique to the mixed habitat (i.e. not found in the monodominant forest plots). We found 58 species (no morphotypes) to be common to both forest types.

For size class between 10 cm and 20 cm, there were 47 species (excluding *G. dewevrei*) in the monodominant forests; 140 species in the mixed forests; and 39 species in common for both forest types. For size class between 20 cm and ≤40 cm, there were 23 species and 83 species in the monodominant forests and mixed forests, respectively; with 14 species in common for both forest types. In monodominant forests, 12 mixed-forest species were in common across at least two of the size classes. Furthermore, there were 18 species (excluding *G. dewevrei*) with individuals of dbh≥40 cm in the monodominant forests, and 51 species (with individuals of dbh≥40 cm) in the mixed forests. Ten species – *Alstonia boonei, Celtis tessmannii, Celtis zenkeri, Erythrophleum suaveolens, Gambeya lacourtiana, Guarea thompsonii, Ongokea gore, Pentaclethra macrophylla, Petersianthus macrocarpus* and *Polyalthia suaveolens* –had large individuals (dbh≥40 cm) in both forest types. Thus, there were species (e.g., *Pentaclethra macrophylla* and *Polyalthia suaveolens*) in common at all size classes, suggesting successful co-occurrence, and that some species were competing with *G. dewevrei*.

Overall, after eight years (from 2005 to 2013), the monodominant forests gained five new species, while two species originally present were lost. Just 15 stems that were not *G. dewevrei* died (of 13 species), and 25 stems that were not *G. dewevrei* were recruited (of 15 species). In absolute terms, the most dynamic species gained or lost two individuals over the eight year period. For large trees (>40 cm dbh), the number of species remained the same in 2005 and 2013. In total, the number of non *G. dewevrei* stems increased by 10 individuals. For common species (>10 individuals), of the eight species, six gained or lost a single stem, while the two others – *Trichoscypha acuminate* and *Staudtia stipitata* – increased by three stems and decreased by two stems, respectively. These results show the constancy of the presence of non-dominant species and number of stems.

**Figure 2:**
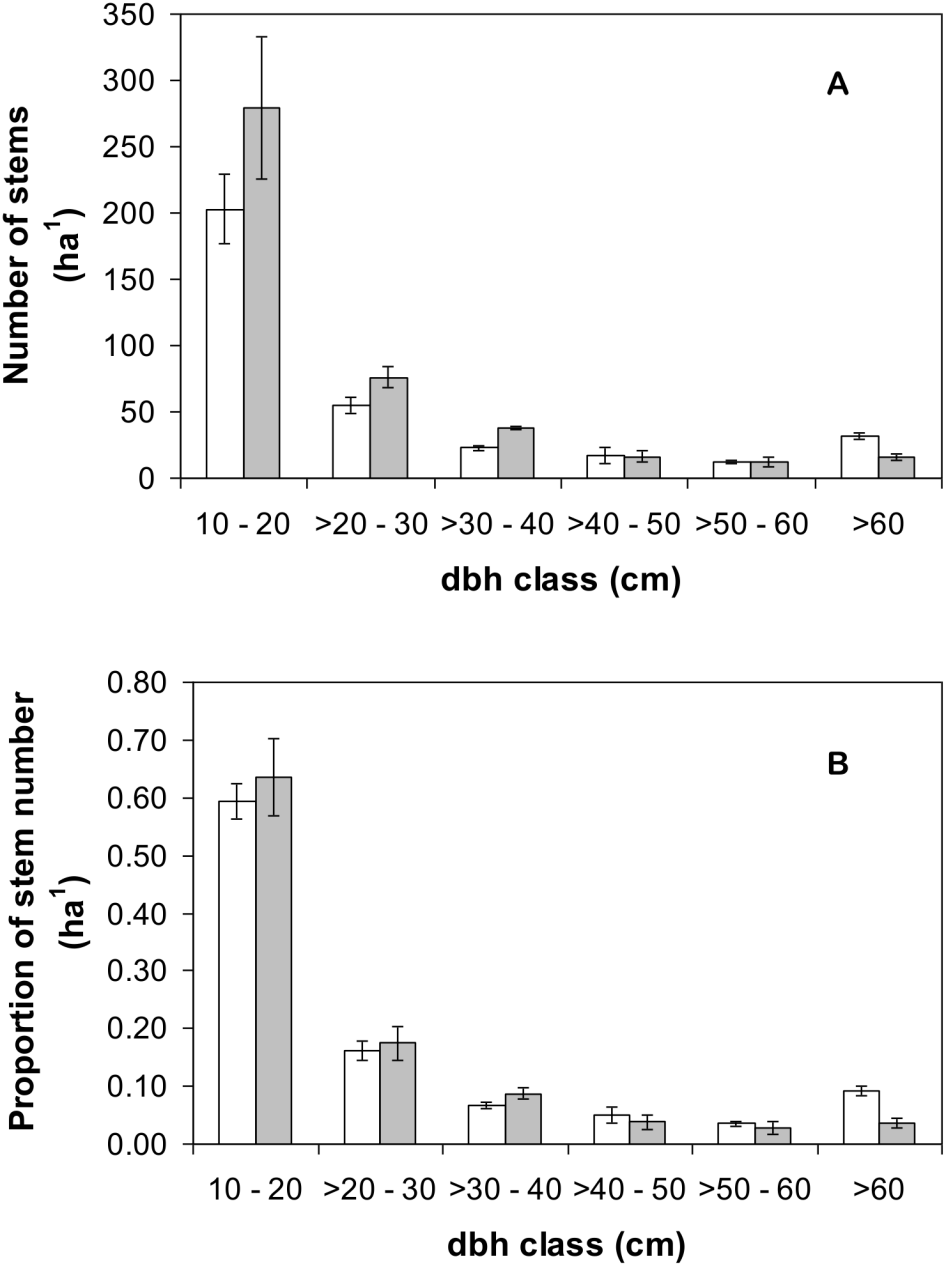
Allocation of number of stems (A) and proportion of stem number (B) in different diameter in breast height (dbh) classes in monodominant *Gilbertiodendron* forests (white bars) and mixed forests (grey bars). Error bars represent 95% confidence intervals.

There are some similarities between the monodominant and mixed forests in terms of their structures. Most of the individual stems with dbh≥10 cm in both forest types were in the dbh class of 10–20 cm (Fig. 2). Both forest systems had their number of stems decreased with increasing size classes (Fig. 2). Nevertheless, the structure of the monodominant forests is distinct from that of the mixed forest: the mean number of stems per hectare was less in the monodominant forests, with less stems in monodominant forests in size-classes <40 cm dbh, compared to the mixed forests, and more stems >40 cm dbh (Fig. 2a). The monodominant forests also had a greater proportion of trees of dbh>60 cm and lower proportion of trees of dbh>30–40 cm (Fig. 2b; these differences were significant because the 95% confident intervals of the two forest types did not overlap for these size classes).

**Figure 3:**
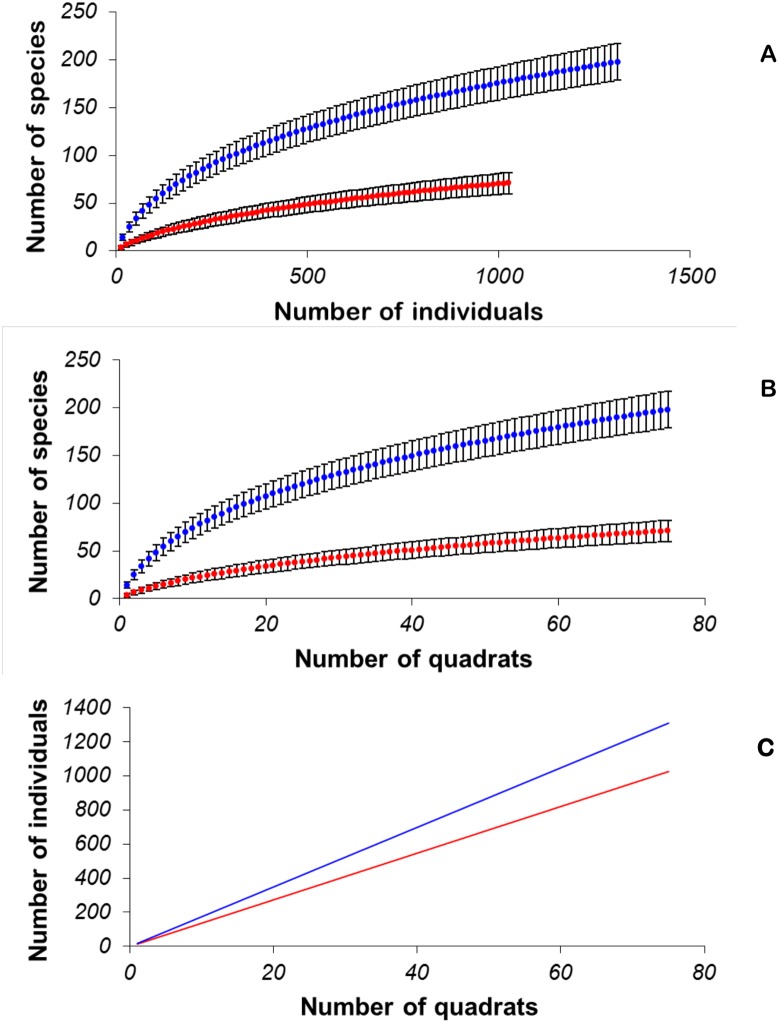
Sample-based rarefaction curves of the tree communities found in monodominant *Gilbertiodendron* forests (red dots and line) and mixed forests (blue dots and line) comparing number of species (A), species density (B) and population density (C). Error bars represent 95% confidence interval.

All calculated metrics showed that the monodominant forest had lower estimated species diversity values than the mixed forest (Table 1). The mean forest tree species richness estimates from the nine estimators were 113.52 for the monodominant forest and 308.39 for mixed forest. The mean number of species recorded per quadrat (i.e., species density) among the vegetation types were 3.73±0.40 and 13.97±0.82 for the monodominant forest and the mixed forest, respectively (for species richness per ha, see Table 2). The rarefaction curves suggested that the monodominant forest has lower tree species richness, tree species density, and population density compared to the mixed forest (Fig. 3, a–c). In line with these results, the monodominant forest had a lower Fisher’s α value than the mixed forest (Table 2). In addition, Hill number of the monodominant forests (98.87±8.23) was significantly lower than that of the mixed forests (263.24±11.90).

**Table 1:** Nonparametric species richness estimations of the monodominant *Gilbertiodendron* forest and the adjacent mixed forest.

Parameters*	Monodominant forest		Mixed forest	
n	75		75	
Sp_obs_	71		198	
Ind_obs_	1026		1310	
ACE	125.85	(0.56)	324.48	(0.61)
ICE	133.91	(0.53)	332.42	(0.60)
Chao1	119.17	(0.60)	391.60	(0.51)
Chao2	129.91	(0.55)	396.03	(0.50)
Jack1	106.52	(0.67)	285.81	(0.69)
Jack2	131.00	(0.54)	353.24	(0.56)
Bootstrap	86.10	(0.82)	234.38	(0.84)
MMRuns	94.81	(0.75)	229.84	(0.86)
MMMean	94.42	(0.75)	227.74	(0.87)
Mean	113.52		308.39	

*A total of 211 tree species and morphotypes were recorded from plot surveys (a total of 3 ha for each forest type). *n* represents sample size (number of 20 m×20 m quadrats); Sp_obs_ and Ind_obs_ represent total number of species and individuals observed, respectively. ACE, ICE, Chao1, Chao2, Jack1, Jack2, Bootstrap, MMRuns and MMMeans are nonparametric species estimators. Number in parentheses represents the proportion of the estimated species richness that was observed.

**Table 2:** Summary of information on forest types including number of tree species, dominance of *Gilbertiodendron dewevrei* and Fisher’s alpha index.

Monodominant forest				Mixed forest			
Plot	Number of treespecies	Dominance(%)	Fisher’sAlpha	Plot	Number oftree species	Dominance (%)	Fisher’s Alpha
G1	33	73	9.37	M1	107	0	45.65
G2	35	85	9.71	M2	130	0	59.19
G3	39	97	10.98	M3	104	0	45.13
Mean:	35.7±3.5	85.0±13.6	10.02±0.96		113.7±16.1	0	49.99±9.02

The values for each forest type are the average from 3 plots, with 95% confidence intervals at *P* = 0.05. The pairs were G1-M1; G2-M2; and G3-M3. Dominance refers to the dominance of *G. dewevrei* in terms of above-ground biomass measuring in 2005. The standard Afritron (http://www.geog.leeds.ac.uk/projects/afritron/) codes of these plots are as follows: G1 (DJK-02), G2 (DJK-03), G3 (DJK-01), M1 (DJK-05), M2 (DJK-06), and M3 (DJK-04).

### Life-history Traits and Fitness Proxies

We recorded 70 tree species (excluding *G. dewevrei*) in the monodominant forests of which 58 species were also found in the mixed forest plots (29.3% of the 198 tree species and morphotypes occurring in mixed forest). These non-dominant species in the monodominant systems were represented by 192 individual stems (18.9% of individual stems recorded in the three monodominant forest plots). At the species level, 23 (35%) non-dominant species in the monodominant forest were considered to be locally common (≥1 stem ha^−1^) and 47 species to be rare (<1 stem ha^−1^) (see Table S1). Only eight common non-dominant species in the monodominant forest had 10 or more individuals recorded within the three 1 ha plots (i.e., ≥3.3 stems ha^−1^): *Angylocalyx pynaerthii* (5.0 stems ha^−1^); *Carapa procera* (4.7 stems ha^−1^); *Desbordesia glaucescens* (4.3 stems ha^−1^); *Staudtia stipitata* (4.0 stems ha^−1^); *Pentaclethra macrophylla* (3.7 stems ha^−1^); *Strombosia pustulata* (3.7 stems ha^−1^); *Trichoscypha acuminata* (3.7 stems ha^−1^); and *Mammea africana* (3.3 stems ha^−1^).

The univariate analysis results indicated that high wood density, high relative abundance in adjacent mixed forest and tolerance of low light levels were significant (*P*<0.05) correlates of non-dominant tree species that were successfully co-existing in monodominant forest (Table 3). Neither tree stature, maximum diameter, geographical range nor dispersal method had any significant impact on the probability of species persistence in monodominant forest. Similar results were obtained when the regressions were repeated having each trait as the sole independent variable without family as a covariate. Among these traits, relative abundance in mixed forest (*P*<0.001) was the most important correlate of tree species success likelihood in establishment in the monodominant forest. The mean abundance (±95% confidence intervals) of the species that successfully established in monodominant forest, in the mixed plots was 4.2±1.5 individuals ha^−1^. This was significantly different from that of the species in mixed forest plots that were absent in monodominant forest plots (1.1±0.3 individuals ha^−1^). Locally common species appear to be able to establish in monodominant forest.

**Table 3:** Relationship between traits of mixed-forest tree species and their occurrence in monodominant *Gilbertiodendron* forests.

		As sole variable			With family		
Variable	n	coefficient	*P*	odds ratio(95% CI)	coefficient	*P*	odds ratio(95% CI)
Relative abundance	193	0.10	<0.001	1.11 (1.06–1.16)**	0.10	<0.001	1.11 (1.05–1.16)**
Wood density	187	3.57	0.004	35.59 (3.22–392.88)*	3.78	0.004	43.92 (3.44–560.35)*
Height	148						
medium		0.50	0.365	1.65 (0.56–4.87)	0.39	0.496	1.47 (0.48–4.50)
tall		0.52	0.361	1.68 (0.55–5.09)	0.33	0.578	1.39 (0.44–4.42)
Diameter at breast height	146						
medium		−0.07	0.879	0.93 (0.38–2.28)	−0.18	0.698	0.83 (0.33–2.11)
large		0.59	0.131	1.80 (0.84–3.85)	0.45	0.283	1.57 (0.69–3.55)
Light requirement	145						
shade tolerance		1.86	0.004	6.44 (1.81–22.87)*	1.82	0.006	6.18 (1.68–22.74)*
Geographical distribution	144						
wide		−0.33	0.656	0.72 (0.16–3.12)	−0.42	0.586	0.66 (0.15–2.96)
Family	192						
Legumes		0.38	0.449	1.45 (0.55–3.84)			
Amnonaceae		0.29	0.619	1.33 (0.43–4.15)			
Euphorbiaceae		−0.61	0.200	0.54 (0.21–1.38)			
Meliaceae		0.98	0.148	2.67 (0.71–10.08)			
Rubiaceae		−1.62	0.131	0.20 (0.02–1.62)			
Sapotaceae		−0.12	0.873	0.89 (0.21–3.77)			

The response variables are “absence” (code = 0) and presence (code = 1). N denotes the number of species; CI stands for confidence interval; *P*<0.001 is denoted by ** and *P*<0.05 by *. Complete information is not available for all species.

**Figure 4:**
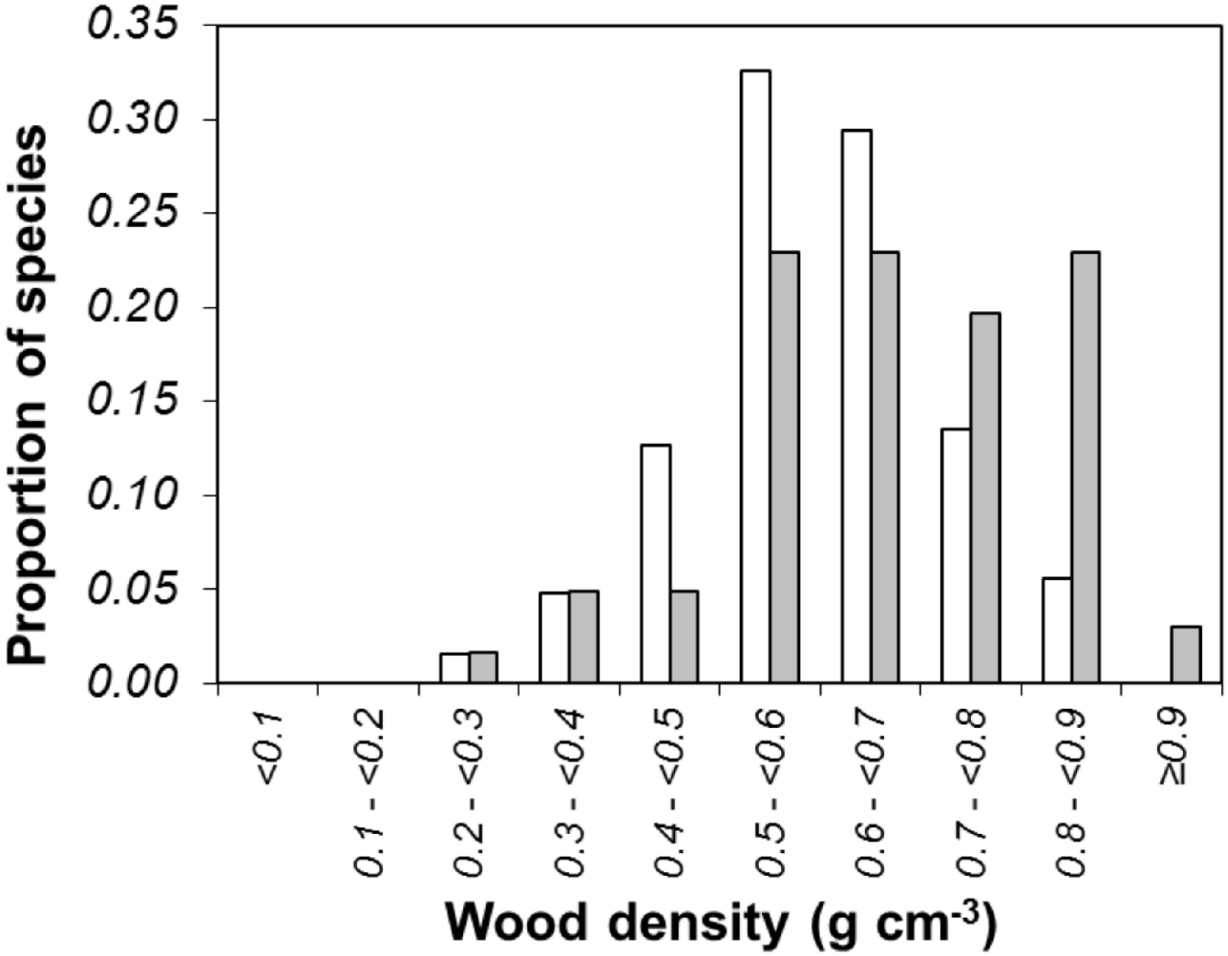
The association of wood density categories and successful mixed-forest species in the monodominant *Gilbertiodendron* forest. The white bars represent the species absent in the monodominant forest and the grey bars represent those present in the monodominant forest.

The mean wood density of all the non-dominant species in the monodominant forest plots was 0.659±0.038 g cm^−3^ and was significantly different from that of the species in the mixed forest plots that were not found in the monodominant forest plots (0.598±0.021 g cm^−3^; 95% confidence intervals of the two forest types did not overlapped). There is an association between species of certain wood density categories and successful establishment in the monodominant forest (Fig. 4). In particular, the species with wood density >0.79 g cm^−3^ were significantly more likely to be established in the monodominant forest (χ^2^ = 15.19, d.f. = 4, *P*<0.01).

Regardless of the influence of family as a covariate, the light requirement is a significant correlate of successful establishment in the monodominant forests. There were only three species (2%) among the non-dominant species in the monodominant forest plots that were pioneer (sensu Swaine and Whitmore [24]), whereas 13% of the species found exclusively in the mixed forest plots were pioneers. The odds of shade intolerance species being successfully established in a monodominant forest are 0.16. But the odds are higher at 0.92 for the shade tolerance species.

**Figure 5:**
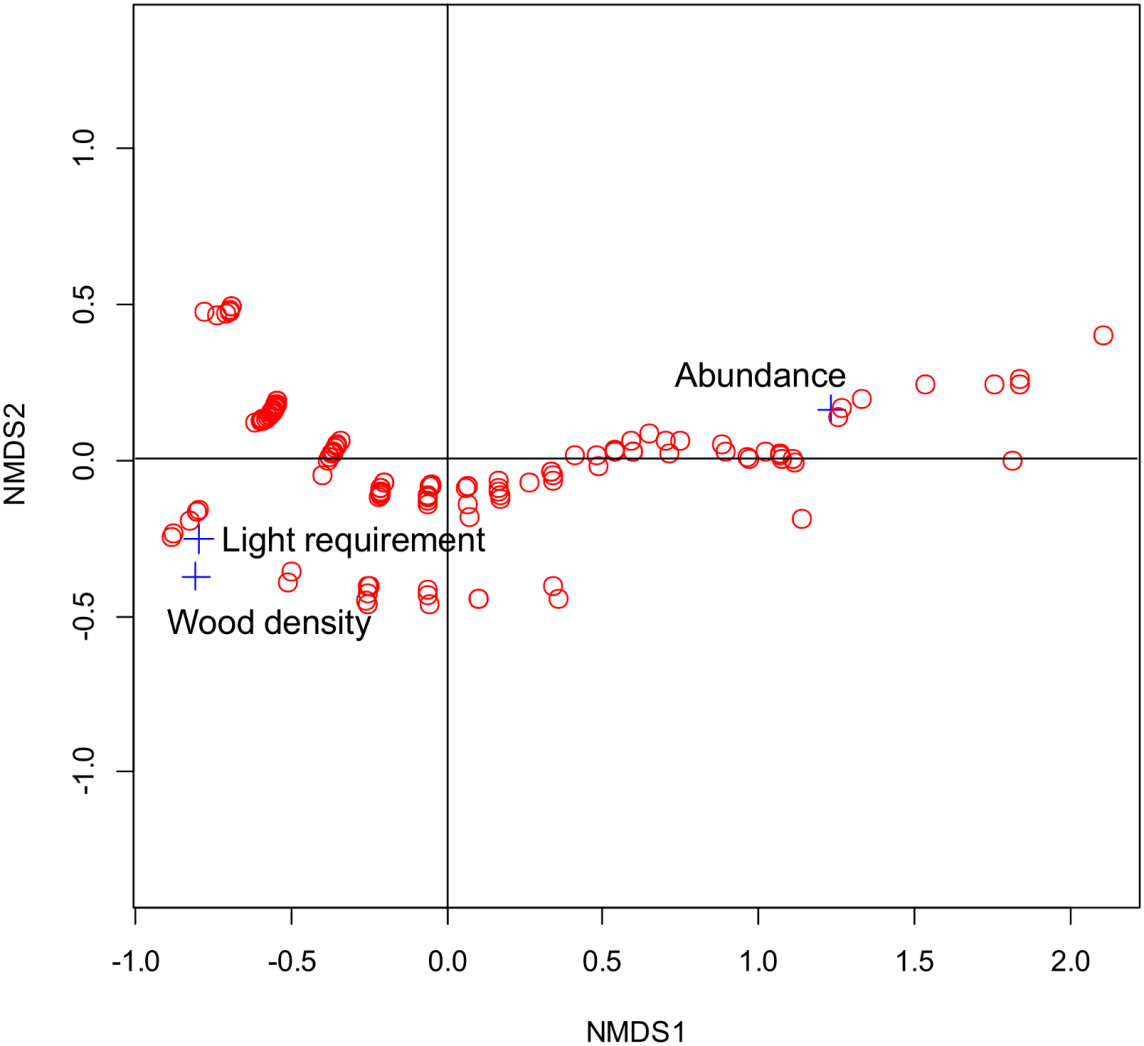
Ordination of wood density, light requirement and relative abundance in the non-metric multidimensional scaling space of tree species. NMD1 and NMDS 2 represent axis 1 and axis 2 of the non-metric multidimensional scaling. The red open circles represent all established tree species (i.e., dbh≥10 cm) found in the three mix-forest plots.

Among the variables retained from the univariate regression analyses, there was evidence of collinearity between wood mass density and light requirement in the NMDS ordination (Fig. 5; the stress value of the final solution on 2 dimensions was 0.04). However, this technique does not indicate if this relationship was significant.

Of the four candidate models generated, based on the permutations of significant variables retained by the univariate analyses, the most parsimonious model selected by AIC_c_ was the one that includes wood density, relative abundance and light requirement (Table 4). This model was at least 49 times more strongly supported by the data than the other simpler variant models (Table 4). The inclusion of both wood density and light requirement does not invalidate this model as collinearity does not affect the results of model fit statistics (i.e. AICc). This is because collinearity influences individual parameter estimates only; it does not affect the overall level of variance accounted for, measured by improvements in fit [33]. Moreover, individual parameters and their standard errors do not affect the global statistics in which redundant variables would simply fail to improve model fit [33]. Hence, as wood mass density and light requirement each improved model fit, there is little evidence of redundancy in the two variables, and indicates that collinearity between these two variables was not a strong relationship.

**Table 4:** Binary regression models for successful non-dominant tree species co-occurrence in monodominant *Gilbertiodendron* forest at Dja Faunal Reserve with corresponding log-likelihood, number of parameter (*K*), Akaike’s information Criterion (AIC_c_) score and Akaike parameter weight (*w*_p_).

Rank	Model	r	log-likelihood	*K*	AICc	ΔAICc	wp
**1**	**abundance + density + shade**	**0.217**	**−79.190**	**4**	**166.66**	**0.00**	**0.98**
2	abundance + shade	0.195	−84.037	3	174.24	7.58	0.02
3	density + shade	0.105	−88.441	3	183.05	16.39	0.00
4	abundance + density	0.189	−101.417	3	209.00	42.34	0.00

ΔAIC_c_ indicates the difference between each model and the best model (lowest ΔAICc; rank 1). Data include 145 tree species sampled in all monodominant and mixed forest plots. Abundance, density and shade refer to fitness proxy and life-history variables: abundance, number of individuals found in the mixed forest plots; density, wood density; and shade, light requirement for seedling establishment. Models are ranked by ΔAIC_c_ and *w*_p_. The best model that has ΔAIC_c_<1 is bolded.

Thus, in this final model, species from the mixed forest with higher wood density and relative abundance, and lower light requirement had a higher probability of establishment in the monodominant forest. The coefficient and odds ratio for wood density (Table 5) express the effect of an increase of 1 g.cm^−3^ in wood density when the other variables in the analyses remain unchanged. However, it is more meaningful if a more realistic specific wood density difference is stated. Thus, for an increment of 0.1 g.cm^−3^, the coefficient 3.03 (Table 5) may be multiplied by 0.1 to obtain 0.30 which is the natural log of 1.35 (odds ratio). In other words, for every 0.1 g cm^−3^, the odds of achieving establishment improve by 1.35. For a given wood density and relative abundance in the adjacent mixed forest, non-pioneer species were 5.1 times more likely to establish in the monodominant forests than species that are light demanding (Table 5). Finally, for a given light requirement and wood density, an increment of 1 individual in a 3 ha mixed forest improves the odds of the species achieving establishment in a 3 ha monodominant forest by 1.09 (Table 5).

**Table 5:** Final multiple logistic regression model explaining the establishment of mixed-forest tree species in the monodominant *Gilbertiodendron* forest.

Parameter	Coefficient	*P*	odds ratio (95% CI)
Constant	−4.10	<0.001	
relative abundance	0.08	0.002	1.09 (1.03–1.14)
wood density	3.03	0.033	20.67 (1.28–334.74)
light requirement			
shade tolerance	1.63	0.033	5.11 (1.14–22.96)

Model concordance = 78.7%; n = 140.

## Discussion

In south-eastern Cameroon, we compared species richness among the trees with stem diameters ≥10 cm in monodominant *G. dewevrei* forest, to that of the adjacent mixed forest. For each forest type, we calculated Fisher’s α value for comparison purposes with other African data [38], [39]. Mean Fisher’s α values (±95% confidence interval, n = 3) of trees with dbh≥10 cm, for the monodominant and mixed plots in this study were 10.02±0.96 and 49.99±9.02, respectively. Fisher’s α of trees with dbh≥10 cm in our mixed forest plots (1 ha) was higher than that of mixed forest plots (Fisher’s α: 19.5 and 21.9 based on sampling at the 1 ha scale of two 10 ha plots) at Ituri Forest in the Democratic Republic of the Congo [38], but within the range recorded for a typical African mixed forest (mean Fisher’s α±SD: 40.4±13.8 based on sampling at the 1 ha scale; range = 7.8–66.1 [39]). Although it is not surprising that our monodominant forest plots (1 ha) had lower species diversity than the mixed plots in Africa, they were also less diverse as compared to the monodominant *G. dewevrei* plots at Ituri Forest (Fisher’s α based on trees with dbh≥10 cm: 15.3 and 20.2 based on sampling at the 1 ha scale of two 10 ha plots [38]). Further comparisons with studies of monodominant forests from other regions (e.g. Neotropics) are restricted by limited sampling efforts and the data reported [40].

There are only a handful of monodominant forest sites whereby their tree species diversity was compared to the adjacent mixed forests (e.g., *G. dewevrei* forests at Ituri [3], [7]; *Nothofagus* forests in New Caledonia [40]). Although lower species richness in the monodominant forest shown in our study is in accordance with some studies [40], our results are in contradiction to those of Makana and others (2004) [7]. At Ituri forest plots, Makana and others (2004) found that species richness, based on stem size ≥10 cm in sampling areas of 1 ha, was comparable in the monodominant and mixed forests [7]. This study suggested that most of the richness in monodominant forest is accounted for either by rare species or by species with highly clumped distributions (i.e., high patchiness). However, our results provide evidence that the *G. dewevrei* forests had lower species richness, species density and population density than its adjacent mixed forests in terms of established trees of dbh≥10 cm. The asymptotic smoothed species accumulation curves for the monodominant and mixed forests indicate that these forests were generally adequately sampled. Non-parametric estimators of species richness suggest the same level of completeness for our species inventories within the monodominant forest (53–82% of estimated species detected) and mixed forest (52–87%).

Admittedly, the total sampling area for each forest type in this study was only 3 ha. However, we compared the species richness between the two forest types based on taxon sampling curves that accounts for (1) differences in sampling effort (e.g., number of stems measured) between the forest types and (2) natural levels of sample heterogeneity (i.e., patchiness) in the data [27]. Additionally, all the nine nonparametric species richness estimators unanimously showed that the monodominant forests have lower estimated true species richness. The discrepancy of our findings with those of Makana and others (2004) [7] could be due to the lower diversity in the Ituri mixed forest plots (Fisher’s α based on trees with dbh≥10 cm: 19.5 and 21.9 per hectare based on two 10 ha plots [38]) and hence, a greater number of individuals per species in those plots when the number of stems being approximately equal (number of stems based on trees with dbh≥10 cm in the Ituri mixed forests: 425 stems and 451 stems per hectare based on two 10 ha plots [38]), compared to our mixed forest plots. From this study, we know that a higher relative abundance of the mixed forest species could increase their success in co-occurring with *G. dewevrei*, leading to a larger number of mixed forest species in the Ituri monodominant forests. Our findings suggest that there may be a regional variation in species richness among the *G. dewevrei* forests, and the association of relatively high species richness with monodominance remains inconclusive and equivocal.

In this study, we evaluated each life history trait to identify important correlates for the non-dominant species to establish and attain maturity (i.e., dbh≥10 cm) in the monodominant forest (see Table S2). It has been hypothesized that shorter tree species that are able to complete their life cycles under the shade will be more represented in the monodominant stands [7],[38]. Similarly, slender species (trees with small maximum dbh size) should be able to grow in height at a faster rate and to have better access to light in the closing canopy [41] than those with a larger girth, and therefore may also be more represented in the monodominant stands. However, tree height at maturity was not a significant correlate of establishment probability in the monodominant stands nor was maximum dbh.

More widely distributed species may be better able to exploit a wide range of ecological niches than species with narrow distributions [42]. Although geographical distribution may be an indication of the ability to tolerate different environmental conditions across a wide range of taxonomic groups (e.g., primates [43]; birds [44]), species with wide geographical distribution size was not a significant positive determinant of successful establishment in the monodominant forests. Our results suggest that the more widely distributed species are not inherently more adapted to the monodominant forests.

Tree species in mixed forests that are dependent on biotic agents could enhance the beneficial dispersal of their seeds through reliable disperser visitation to maximize the chances for their seed to be deposited in the monodominant forests. This is on the basis that many of the seed dispersal agents (e.g., primates and hornbills) have large home ranges, thus the probability of their seeds being transported to the monodominant forests is increased. In this study, the biotic dependent dispersal mode had no significant association with the tree species establishment in the monodominant forests. Instead, approximately 15% of the common non-dominant species (i.e., at least one individual per ha) in the monodominant forests were dependent on wind, explosive mechanism or no obvious adaptation.

This may imply that the non-dominating species do not need to rely on long-ranging animal dispersers to enhance their establishment in monodominant forests.

Our results indicate evidence for the importance of relative abundance in mixed forests for the occurrence of mature individuals in the monodominant forests. The presence of a greater number of individuals in mixed forests significantly increased the establishment success of tree species in the adjacent monodominant forests. Species’ relative abundance in the mixed forests was important for their establishment in the adjacent monodominant forests for two possible reasons. First, probability of establishment in the monodominant forests is dependent on the number of propagules released, which in turn depends on the number of individuals in the nearby mixed forests. Success rate for establishment may increase as more propagules are released. Second, more individuals in the mixed forests means that repeated attempts could occur before the species successfully established themselves. An analogy is the classic case of introduction success in exotic New Zealand birds with larger initial population size [45]. Relative abundance that significantly influenced the successful establishments of non-native species is well known from ecological invasion studies [46], [47].

The second most important trait that determined the probability of successful establishment of tree species in the monodominant forests was wood density. Species with higher wood density had a greater probability of establishing in the monodominant forests. However, slow-growing, shade tolerant species with high wood density are very similar to *G. dewevrei* and probably occupy the similar niche as the monodominant species. But wood density being a significant determinant, alongside with light requirement, suggests that high wood density may confer some advantages. One possible benefit is that having heavy wood might reduce the probability of physical damage caused by falling debris under the closed *G. dewevrei* canopy. Wood density is strongly positively correlated to wood strength and stiffness [48], [49]. Due to the denser canopy of *G. dewevrei*, the damage frequencies in monodominant forests may be higher as compared to mixed forests. For example, young saplings in our monodominant plots during the masting year was subjected a total of 3.62 Mg ha^−1^ yr^−1^ of falling large seeds (average dry seed mass = 20.4 g) and branches (1cm–10 cm in diameter) as compared to 2.54 Mg ha^−1^ yr^−1^ in the mixed plots (KSHP, unpublished data). The importance of denser and tougher wood in plants growing under closed canopy is also supported by studies showing that the closed forests have higher damage rates than in more open areas [50]; and wood density is positively related to the sapling survival of forest tree species [51]. The reduction of species richness among stems with dbh>10 cm in the monodominant forests compared to species richness including individuals >1 cm dbh by Makana and others (2004) [38] might be due to the high mortality among smaller trees (dbh<10 cm) caused by the falling debris of *G. dewevrei*. Lending credence to this speculation, studies have shown that although falling debris can cause high mortality in saplings [52], physical damage has become less important once saplings have reached a dbh>10 cm [53]. Therefore, having stronger wood may be an important factor that may confer an advantage for a species to grow beyond 10 cm in the monodominant forests.

The third important trait for the species establishment in monodominant forests is their light requirement for seedling establishment. Pioneer species that demand full sunlight for seedling establishment [54] will be affected under *G. dewevrei* forest shade. This is because the monodominant forests have lower light penetration in the understorey than the mixed forest due to the relatively homogeneous, closely packed and deep crowns of *G. dewevrei* [5], [7], [38]. Our study also shows that *G. dewevrei* forests had greater numbers of very large trees (dbh>60 cm) and these trees could cast deep shade. Moreover, pioneer species may fail to establish to maturity under the monodominant canopy because the deaths of large *G. dewevrei* due to natural mortality are relatively uncommon and they tend to die while remaining standing, therefore not providing large gaps in the forest canopy for regeneration. Thus, fewer large gaps are present to provide direct sunlight for the light demanding species to recruit to maturity (KSHP, personal observations). All the common species were non-pioneers. Examples of these non-dominant species that can establish under the forest shade are *Angylocalyx pynaerthii, Carapa procera*, *Desbordesia glaucescens*, *Staudtia stipitata*, *Pentaclethra macrophylla*, *Strombosia pustulata*, *Trichoscypha acuminata* and *Mammea africana.*

Our dataset of species composition of the monodominant and mixed forests is a valuable resource for studying the factors underlying successful co-existence of tree species in monodominant forests. However, the value of this dataset may be limited by the lack of information on tree species with dbh<10 cm in the monodominant forests. On the other hand, identifying species richness and the characteristics of the established non-dominant tree assembly may be more ecologically meaningful because the young saplings that fail to complete their life cycles might have limited contributions or influence on the functioning of the ecosystem.

### Implications for Forest Invasibility

In this study, we assume one particular trajectory of past and current vegetation dynamics, i.e. tree species which colonize and establish themselves in monodominant patches of *G. dewevrei* from their source populations in the adjacent mixed forest. One could also argue for another possible trajectory of community assembly: *G. dewevrei* established in the mixed forest and, by expanding vigorously, outcompeted many of the resident species that did not compete successfully. However, the former trajectory is more likely the case because we have observed a higher diversity of sapling species than canopy (i.e. established) species in our monodominant plots. This was only possible by dispersal from the source populations in the adjacent mixed forests. Furthermore, when we analysed the survivorship and recruitment of non-dominant stems after eight years, we saw that richness was not declining, the number of non-dominant stems was not declining, and nor was the number of species or stems of the largest size classes (>40 cm dbh). This is all consistent with ongoing establishment and co-occurrence of some non-dominant species in monodominant forests.

The establishment potential of mixed-forest tree species in classical monodominant forests is poorly known in the tropical regions. We encountered seven mixed-forest species occurring with a total of more than 10 large individuals in our monodominant plots. These findings strongly indicate that some mixed-forest tree species can successfully establish into the monodominant stands of *G. dewevrei* from south-east Cameroon. Furthermore, when comparing mixed-forest tree species that were present in the monodominant forests with those that were absent, the mix-forest species in the monodominant stands have higher number of individuals in their original habitat, higher wood density and lower light requirement of seedling establishment. This may imply that these traits could be some of the attributes that an invasive ‘alien’ possesses in order to exploit an undisturbed, closed environment.

However, this conclusion is not completely consistent with other research that documented the traits of invasive aliens. For example, contrary to our finding that lower light requirement is associated with invasibility in a closed-canopy forest, Tanentzap and Bazely (2009) found that high light availability is important for the establishment of invasive species in a forest [55]. Also, species in the family Pinaceae – which many are invasive [56] – have low wood density that allows them to grow rapidly in height and compete for light when gaps occur in a forest [57]. Therefore, we propose that (1) invasive aliens exploiting an undisturbed forest may not exhibit the same set of traits as those successful aliens in an open, disturbed habitat; and (2) aliens that invade undisturbed and disturbed forests may exhibit different successional dynamics. Hence, aliens exploiting undisturbed, closed systems may be distinguishable from those exploiting disturbed, open habitats. Nevertheless, alien species (‘mixed-forest tree species’) invading undisturbed forests (‘monodominant forests’) are likely to have the general suite of traits exhibited by the native species of these forests (‘*Gilbertiodendron dewevrei*’).

Ecologists have tended to view that a vacant niche (e.g., an open gap) is essential for a successful invasion process [55]. Despite invasions are often associated with disturbed systems [58], our study suggests that the tropical mature forests with low exogenous disturbance regimes over long periods (i.e., classical monodominant forests) are ‘invasible’. Therefore, in search for the distinction between the aliens exploiting disturbed and undisturbed systems may be useful in the context of protecting the remaining relatively pristine forests from invasive species. We urgently need a better and more integrated understanding of the invasion processes occurring in closed-canopy habitats. Because our understanding of biological invasion has relied upon studies focusing on aliens in disturbed environments, our findings may bring new insights for predicting the important traits of potential aliens in tropical mature forests, and show strong evidence that tropical mature forests could also be susceptible to biological invasion. We hope our findings will spur more research to identify traits and life histories of potentially invasive species that threaten relatively undisturbed tropical forests.

## Supporting Information

Table S1 **List of tree species and their abundance**. List of tree species and their total number of individuals (dbh>10 cm) found in three 1 ha plots within two forest types, monodominant *Gilbertiodendron* forest (mono) and mixed forest (mix). Species in bold had no individual with dbh≥10 cm in mixed forest plots.
(DOCX)

Table S2 **Traits and life histories of all tree species.** Life-history trait data set for the 193 species at the Dja Faunal Reserve based on published literature (e.g., Lewis *et al*. 2009; Sonké 2004; Poorter et al. 2003; van Gemerden *et al*. 2003), herbarium specimens and personal observations. The relative abundance of each species in the mixed forests was based on the three mixed forest plots. Wood mass density is defined as dry wood mass/green wood volume (g cm^−3^) and is compiled from Lewis *et al*. (2009). Species were classified according to maximum stature in three classes: large trees (>30 m tall), medium trees (10–30 m tall) and small trees (<10 m tall). Species were placed on the basis of maximum dbh in three classes: large diameter (>100 cm in dbh), medium diameter (50 cm–100 cm) and small diameter (<50 cm). Each species is classified in one of two categories according to its fruit/seed dispersal mode (biotic and non-biotic dependent). Each species was classified into one of two categories according to its ecological guild in terms of light requirement (pioneer [e.g., light-demanders that require high light level for seedling establishment], and non-pioneer [shade-bearers that are capable of seedling establishment under forest shade, though some shade-bears may need higher light level at later stage of life]), and each was grouped according to its geographical distribution (narrow, i.e., species endemic to lower-Guinea-Congolean biogeographical region; and wide that includes species which were not endemic to the region).
(DOCX)
