# Supplementary material for: Mixed-Forest Species Establishment in a Monodominant Forest in Central Africa: Implications for Tropical Forest Invasibility
Source: PLoS One. 2014 May 20;9(5):e97585. doi: 10.1371/journal.pone.0097585 (PMC4028239; doi:10.1371/journal.pone.0097585)
Supplement: Table S1 — List of tree species and their abundance. List of tree species and their total number of individuals (dbh>10 cm) found in three 1 ha plots within two forest types, monodominant Gilbertiodendron forest (mono) and mixed forest (mix). Species in bold had no individual with dbh≥10 cm in mixed forest plots. (DOCX) [file pone.0097585.s001.docx]

| \| Family \| Name \| Forest types \| \| \| --- \| --- \| --- \| --- \| \|  \|  \| mono \| mix \| \| Clusiaceae \| *Allanblackia floribunda* Oliv. \| 0 \| 4 \| \| Apocynaceae \| *Alstonia boonei* De Wild. \| 1 \| 7 \| \| Fabaceae \| *Amphimas pterocarpoides* Harms \| 0 \| 4 \| \| Fabaceae \| *Angylocalyx pynaerthii* De Wild. \| 15 \| 6 \| \| Annonaceae \| *Anonidium mannii* (Oliv.) Engl. & Diels \| 1 \| 95 \| \| Rhizophoraceae \| ***Anopyxis klaineana* (Pierre) Engl.** \| 1 \| 0 \| \| Fabaceae \| *Anthonotha cladantha* (Harms) Léonard \| 1 \| 1 \| \| Fabaceae \| *Anthonotha macrophylla* Pal. Beauv. \| 0 \| 9 \| \| Eurphorbiaceae \| Antidesma sp. A \| 0 \| 1 \| \| Sapotaceae \| ***Baillonella toxisperma* Pierre** \| 2 \| 0 \| \| Lauraceae \| *Beilschmiedia* sp. A \| 0 \| 1 \| \| Lauraceae \| *Beilschmiedia* sp. B \| 0 \| 1 \| \| Sapindaceae \| *Blighia sapida* Konig \| 3 \| 12 \| \| Sapindaceae \| *Blighia welwitschii* (Hiern) Radlk. \| 1 \| 4 \| \| Rubiaceae \| *Brenania brieyi* (De Wild.) Petit \| 0 \| 2 \| \| Fabaceae \| *Calpocalyx dinklagei* Harms \| 0 \| 1 \| \| Burseraceae \| *Canarium schweinfurthii* Engl. \| 0 \| 1 \| \| Rubiaceae \| Canthium sp. A \| 0 \| 1 \| \| Melicaceae \| *Carapa procera* DC. \| 14 \| 66 \| \| Melicaceae \| Carapa sp. A \| 0 \| 4 \| \| Bombaceae \| *Ceiba pentandra* (L.) Gaertn. \| 0 \| 1 \| \| Ulmaceae \| *Celtis tessmannii* De Wild. \| 1 \| 15 \| \| Ulmaceae \| *Celtis zenkeri* Engl. \| 1 \| 23 \| \| Eurphorbiaceae \| *Centroplacus glaucinus* Pierre \| 3 \| 24 \| \| Sapindaceae \| *Chytranthus* sp. A \| 0 \| 1 \| \| Sapindaceae \| *Chytranthus* sp. B \| 0 \| 1 \| \| Sapindaceae \| *Chytranthus* sp. C \| 0 \| 1 \| \| Sapindaceae \| *Chytranthus* sp. D \| 0 \| 1 \| \| Sapindaceae \| *Chytranthus* sp. E \| 0 \| 1 \| \| Sapindaceae \| *Chytranthus* sp. F \| 0 \| 1 \| \| Annonaceae \| *Cleistopholis glauca* Pierre ex Engl. & Diels \| 0 \| 5 \| \| Annonaceae \| *Cleistopholis patens* (Benth.) Engl. & Diels \| 0 \| 1 \| \| Myristicaceae \| *Coelocaryon preussii* Warb. \| 0 \| 6 \| \| Rubiaceae \| *Coffea* sp. A \| 0 \| 1 \| \| Sterculiaceae \| *Cola acuminata* (P.Beauv.) Schott & Engl. \| 2 \| 6 \| \| Sterculiaceae \| *Cola lateritia* K.Schum \| 0 \| 8 \| \| Fabaceae \| *Cylicodiscus gabunensis* Harms \| 0 \| 2 \| \| Burseraceae \| *Dacryodes edulis* (G.Don) H.J.Lam \| 0 \| 7 \| \| Burseraceae \| *Dacryodes sp. A* \| 0 \| 1 \| \| Irvingiaceae \| *Desbordesia glaucescens* (Engl.) Van Thiegh. \| 13 \| 13 \| \| Tiliaceae \| *Desplatsia chrysochlamys* (Mildbr. & Burrey) Mildbr. \| 0 \| 4 \| \| Tiliaceae \| *Desplatsia dewevrei* (De Wild. & Th.Dur.) Burrey \| 0 \| 11 \| \| Fabaceae \| *Dialium guineense* Wild. \| 3 \| 5 \| \| Ebenaceae \| *Diospyros crassiflora* Hiern \| 1 \| 4 \| \| Ebenaceae \| *Diospyros hoyleana* F.White \| 0 \| 5 \| \| Eurphorbiaceae \| *Discoglypremna caloneura* (Pax) Prain \| 0 \| 3 \| \| Fabaceae \| *Distemonanthus benthamianus* Baill. \| 0 \| 31 \| \| Sapotaceae \| *Donella pruniformis* (Pierre ex Engl.) Aubr. & Pellegr. \| 0 \| 2 \| \| Eurphorbiaceae \| *Drypetes chevalieri* Beille \| 1 \| 3 \| \| Eurphorbiaceae \| *Drypetes cf similis* \| 0 \| 2 \| \| Eurphorbiaceae \| *Drypetes floribunda* (Müll.Arg.) Hutch. \| 0 \| 2 \| \| Eurphorbiaceae \| ***Drypetes gossweileri* S.Moore** \| 1 \| 0 \| \| Eurphorbiaceae \| *Drypetes ivorensis* Hutch. & Dalziel \| 2 \| 1 \| \| Eurphorbiaceae \| *Drypetes laciniata* (Pax) Hutch. \| 0 \| 3 \| \| Eurphorbiaceae \| *Drypetes* sp. A \| 0 \| 1 \| \| Eurphorbiaceae \| *Drypetes* sp. B \| 0 \| 1 \| \| Eurphorbiaceae \| *Drypetes* sp. C \| 0 \| 1 \| \| Eurphorbiaceae \| *Drypetes* sp. D \| 0 \| 1 \| \| Eurphorbiaceae \| *Drypetes* sp. E \| 0 \| 1 \| \| Eurphorbiaceae \| *Drypetes* sp. F \| 0 \| 1 \| \| Tiliaceae \| *Duboscia macrocarpa* Bocq. \| 2 \| 3 \| \| Annonaceae \| *Enantia chlorantha* Oliv. \| 1 \| 24 \| \| Melicaceae \| *Entandrophragma angolense* (Welw.) C.DC. \| 0 \| 1 \| \| Melicaceae \| *Entandrophragma cylindricum* (Sprague) Sprague \| 1 \| 1 \| \| Sapindaceae \| *Eriocoelum macrocarpum* Gilg ex Radlk. \| 1 \| 23 \| \| Sterculiaceae \| *Eribroma oblongum* (Mast) Pierre ex Germian \| 0 \| 1 \| \| Vochysiaceae \| *Erismadelphus exsul* Mildbr. \| 0 \| 1 \| \| Fabaceae \| *Erythrophleum suaveolens* (Guil. & Perr.) Brenan \| 1 \| 4 \| \| Myrtaceae \| *Eugenia* sp. A \| 0 \| 1 \| \| Myrtaceae \| *Eugenia* sp. B \| 0 \| 1 \| \| Bigononiaceae \| *Fernandoa adolfi-fredirici* Gilg. & Mildbr. \| 1 \| 1 \| \| Moraceae \| *Ficus* sp. A \| 0 \| 1 \| \| Apocynaceae \| *Funtumia africana* (Benth.) Stapf \| 0 \| 1 \| \| Apocynaceae \| *Funtumia elastica* (Preuss) Stapf \| 0 \| 5 \| \| Sapotaceae \| *Gambeya lacourtiana* (De Wild.) Aubr. \| 3 \| 10 \| \| Sapotaceae \| *Gambeya perpulchra* (Mildbr. Ex Hutch & Dalziel) Aubrév & Pellegr. \| 0 \| 1 \| \| Sapotaceae \| *Gambeya* sp. A \| 0 \| 1 \| \| Sapotaceae \| *Gambeya* sp. B \| 0 \| 1 \| \| Clusiaceae \| *Garcinia mannii* Oliv. \| 0 \| 10 \| \| Clusiaceae \| *Garcinia punctata Stapf* \| 0 \| 1 \| \| Fabaceae \| ***Gilbertiodendron dewevrei* (de Wild.) Léonard** \| 805 \| 0 \| \| Melicaceae \| *Guarea cedrata* (A.Chev.) Pellegr. \| 1 \| 8 \| \| Melicaceae \| *Guarea thompsonii* Sprague & Hutch. \| 1 \| 14 \| \| Olacaeae \| *Heisteria trillesiana* Pierre \| 1 \| 18 \| \| Olacaeae \| *Heisteria zimmereri* Engl. \| 0 \| 1 \| \| Annonaceae \| *Hexalobus crispiflorus* A.Rich. \| 0 \| 3 \| \| Flacourtiaceae \| *Homalium dolichophyllum* Gilg \| 0 \| 1 \| \| Flacourtiaceae \| *Homalium letestui* Pellegr. \| 0 \| 1 \| \| Fabaceae \| *Hylodendron gabunense* Taubert \| 1 \| 6 \| \| Eurphorbiaceae \| *Hymenocardia lyrata* Tul. \| 0 \| 4 \| \| Eurphorbiaceae \| *Hymenocardia* sp. A \| 0 \| 2 \| \| Irvingiaceae \| *Irvingia gabonensis* (Aurey-Lecomte ex O'Rorke) Baill. \| 2 \| 5 \| \| Irvingiaceae \| *Irvingia grandifolia* (Engl.) Engl. \| 2 \| 2 \| \| Irvingiaceae \| *Irvingia robur* Mildbr. \| 2 \| 1 \| \| Fabaceae \| *Isomacrolobium* sp. A \| 0 \| 3 \| \| Eurphorbiaceae \| *Keayodendron bridelioides* (Hutch. & Dalz.) Léandri \| 0 \| 4 \| \| Eurphorbiaceae \| *Klaineanthus gaboniae* Pierre ex Prain \| 0 \| 3 \| \| Eurphorbiaceae \| *Klaineanthus* sp. A \| 0 \| 1 \| \| Irvingiaceae \| *Klainedoxa gabonensis* Pierre \| 3 \| 7 \| \| Anacardiaceae \| *Lannea welwitschii* (Hiern) Engl. \| 0 \| 1 \| \| Rhamnaceae \| ***Lasiodiscus mannii* Hook.f.** \| 1 \| 0 \| \| Lepidobotryaceae \| *Lepidobotrys staudtii* Engl. \| 1 \| 12 \| \| Chrysobalanaceae \| *Licania elaeosperma* (Mildbr.) Prance & F.White \| 0 \| 1 \| \| Melicaceae \| *Lovoa trichilioides* Harms \| 1 \| 3 \| \| Eurphorbiaceae \| *Macaranga* sp. A \| 0 \| 3 \| \| Eurphorbiaceae \| *Macaranga spinosa* Müll.Arg. \| 0 \| 4 \| \| Eurphorbiaceae \| *Maesobotrya dusenii* (Pax) Hutch. \| 0 \| 6 \| \| Rhamnaceae \| *Maesopsis eminii* Engl. \| 0 \| 2 \| \| Chrysobalanaceae \| *Magnistipula* sp. A \| 0 \| 1 \| \| Clusiaceae \| *Mammea africana* Sabine \| 10 \| 1 \| \| Sapotaceae \| *Manilkara letouzeyi* Aubr. \| 0 \| 1 \| \| Eurphorbiaceae \| ***Mareyopsis longifolia* (Pax) Pax & Hoffm.** \| 3 \| 0 \| \| Chrysobalanaceae \| *Maranthes glabra* (Oliv.) Prance \| 0 \| 2 \| \| Chrysobalanaceae \| *Maranthes* sp. A \| 0 \| 1 \| \| Eurphorbiaceae \| *Margaritaria discoidea* (Baill.) Webster \| 0 \| 3 \| \| Melastomataceae \| *Memecylon amshoffiae* Jacq.-Fél. \| 0 \| 1 \| \| Pandaceae \| *Microdesmis puberula* Hook.f. ex Planch. \| 0 \| 2 \| \| Moraceae \| *Milicia excelsa* (Welw.) Berg. \| 0 \| 1 \| \| Fabaceae \| *Millettia laurentii* De Wild. \| 0 \| 1 \| \| Moraceae \| *Musanga cecropioides* R.Br. \| 0 \| 1 \| \| Moraceae \| *Myrianthus arboreus* P.Beauv. \| 0 \| 21 \| \| Rubiaceae \| *Nauclea diderrichii* (De Wild.) Merril \| 0 \| 3 \| \| Simaroubaceae \| *Odyendya gabonensis* (Pierre) Engl. \| 5 \| 2 \| \| Flacourtiaceae \| *Oncoba glauca* (P.Beauv.) Planch. \| 0 \| 29 \| \| Olacaeae \| *Ongokea gore* Pierre \| 3 \| 2 \| \| Annonaceae \| *Pachypodanthium staudtii* (Engl. & Diels) \| 0 \| 3 \| \| Sapindaceae \| *Pancovia pedicellaris* Radlk. & Gilg. \| 0 \| 1 \| \| Pandaceae \| *Panda oleosa* Pierre \| 1 \| 15 \| \| Fabaceae \| *Parkia bicolor* A.Chev. \| 0 \| 5 \| \| Rubiaceae \| *Pauridiantha floribunda* (K.Schum. Ex K.Krause) Bremek. \| 0 \| 2 \| \| Rubiaceae \| *Pausinystalia macroceras* (K.Schum.) Pierre \| 0 \| 1 \| \| Fabaceae \| *Pentaclethra macrophylla* Benth. \| 11 \| 45 \| \| Lecythidaceae \| *Petersianthus macrocarpus* (Beauv.) Liben \| 1 \| 60 \| \| Apocynaceae \| *Picralima nitida* (Stapf) Th.Dur. \| 0 \| 3 \| \| Mimosaceae \| *Piptadeniastrum africanum* (Hook.f.) Brenan \| 0 \| 6 \| \| Eurphorbiaceae \| ***Plagiostyles africana* (Müll.Arg.) Prain** \| 1 \| 0 \| \| Annonaceae \| *Polyalthia suaveolens* Engl. & Diels \| 7 \| 67 \| \| Anacardiaceae \| *Pseudospondias microcarpa* (A.Rich.) Engl. \| 0 \| 4 \| \| Combretaceae \| *Pteleopsis hylodendron* Mildbr. \| 0 \| 1 \| \| Fabaceae \| *Pterocarpus mildbraedii* Harms \| 0 \| 4 \| \| Fabaceae \| *Pterocarpus soyauxii* Taub. \| 2 \| 11 \| \| Myristicaceae \| *Pycnanthus angolensis* (Welw.) Exell \| 0 \| 5 \| \| Apocynaceae \| *Rauvolfia vomitoria* Afz. \| 0 \| 3 \| \| Ochnaceae \| *Rhabdophyllum* sp. A \| 0 \| 1 \| \| Ochnaceae \| *Rhabdophyllum* sp. B \| 0 \| 1 \| \| Ochnaceae \| *Rhabdophyllum* sp. C \| 0 \| 2 \| \| Ochnaceae \| *Rhabdophyllum* sp. D \| 1 \| 0 \| \| Violaceae \| *Rinorea oblongifolia* (C.H.Wright) Marquand ex Chipp \| 0 \| 3 \| \| Violaceae \| *Rinorea welwitschii* (Oliv.) Kuntze \| 0 \| 1 \| \| Violaceae \| ***Rinorea* sp. A** \| 1 \| 0 \| \| Rubiaceae \| *Rothmannia lujae* (De Wild.) Keay \| 2 \| 20 \| \| Burseraceae \| *Santiria trimera* (Oliv.) Aubrév. \| 7 \| 30 \| \| Flacourtiaceae \| *Scottellia* sp. A \| 0 \| 1 \| \| Anacardiaceae \| *Sorindeia grandifolia* Engl. \| 3 \| 18 \| \| Anacardiaceae \| *Sorindeia mildbraedii* Engl. & Brehmer \| 2 \| 12 \| \| Myristicaceae \| *Staudtia stipitata* Warb. \| 12 \| 8 \| \| Sterculiaceae \| *Sterculia tragacantha* Lindl. \| 0 \| 1 \| \| Olacaeae \| *Strombosia grandifolia* Hook.f. \| 5 \| 5 \| \| Olacaeae \| *Strombosia pustulata* Oliv. \| 11 \| 3 \| \| Olacaeae \| *Strombosia zenkeri* Engl. \| 0 \| 1 \| \| Olacaeae \| *Strombosiopsis tetrandra* Engl. \| 3 \| 8 \| \| Clusiaceae \| *Symphonia globulifera* L.F. \| 0 \| 2 \| \| Sapotaceae \| *Synsepalum dulcificum* (Schum.) Baill. \| 1 \| 11 \| \| Myrtaceae \| *Syzygium rowlandii* (Engl.) Mildbr. \| 0 \| 4 \| \| Apocynaceae \| *Tabernaemontana crassa* Benth. \| 2 \| 54 \| \| Fabaceae \| *Tessmannia africana* Harms \| 2 \| 1 \| \| Fabaceae \| ***Tessmannia anomala* (Micheli) Harms** \| 4 \| 0 \| \| Fabaceae \| *Tetrapleura tetraptera* (Schum. & Thonn.) Taub. \| 0 \| 3 \| \| Moraceae \| *Treculia africana* Desc. \| 3 \| 1 \| \| Rubiaceae \| *Tricalysia discolor* Brenan \| 0 \| 2 \| \| Rubiaceae \| *Tricalysia* sp. A \| 0 \| 1 \| \| Rubiaceae \| *Tricalysia* sp. B \| 0 \| 1 \| \| Melicaceae \| *Trichilia prieuriana* A.Juss. \| 0 \| 1 \| \| Melicaceae \| *Trichilia rubescens* Oliv. \| 0 \| 12 \| \| Melicaceae \| *Trichilia welwitschii* C.DC. \| 1 \| 3 \| \| Anacardiaceae \| *Trichoscypha acuminata* Engl. \| 11 \| 22 \| \| Anacardiaceae \| *Trichoscypha arborea* (A.Chev.) A.Chev. \| 0 \| 1 \| \| Sapotaceae \| *Tridesmostemon omphalocarpoides* Engl. \| 0 \| 2 \| \| Moraceae \| *Trilepisium madagascariense DC.* \| 0 \| 1 \| \| Eurphorbiaceae \| *Uapaca acuminata* (Hutch.) Pax & Hoffm. \| 1 \| 20 \| \| Eurphorbiaceae \| *Uapaca guineensis* Müll.Argr. \| 0 \| 23 \| \| Eurphorbiaceae \| *Uapaca paluosa* Aubrév. & Léandri \| 0 \| 34 \| \| Eurphorbiaceae \| *Uapaca vanhoutei De Wild.* \| 0 \| 2 \| \| Verbenaceae \| *Vitex cienkowskii* Kotschy & Peyr. \| 0 \| 9 \| \| Verbenaceae \| *Vitex grandifolia* Gürke \| 0 \| 1 \| \| Annonaceae \| *Xylopia aethiopica* (Dun.) A.Rich. \| 0 \| 2 \| \| Annonaceae \| *Xylopia hypolampra* Mildbr. \| 1 \| 3 \| \| Annonaceae \| ***Xylopia parviflora* (A.Rich.) Benth.** \| 1 \| 0 \| \| Annonaceae \| *Xylopia quintasii* Engl. & Diels \| 1 \| 14 \| \| Annonaceae \| *Xylopia staudtii* Engl. & Diels \| 0 \| 1 \| \| Annonaceae \| *Xylopia* sp. A \| 0 \| 1 \| \| Annonaceae \| *Xylopia* sp. B \| 0 \| 1 \| \| Rutaceae \| *Zanthoxylum gilletii* (De Wild.) Waterman \| 0 \| 2 \| \| Rutaceae \| *Zanthoxylum heitzii* (Aubr. & Pellegr.) Waterman \| 0 \| 1 \| \|  \| Unidentified sp. A \| 0 \| 1 \| \|  \| Unidentified sp. B \| 0 \| 1 \| \|  \| Unidentified sp. C \| 0 \| 1 \| \|  \| Unidentified sp. D \| 0 \| 1 \| \|  \| Unidentified sp. E \| 0 \| 1 \| \|  \| Unidentified sp. F \| 0 \| 1 \| \|  \| Unidentified sp. G \| 0 \| 1 \| \|  \| Unidentified sp. H \| 0 \| 1 \| \|  \| Unidentified sp. I \| 0 \| 1 \| \|  \| Unidentified sp. J \| 0 \| 1 \| \|  \| Unidentified sp. K \| 0 \| 1 \| \|  \| Unidentified sp. L \| 0 \| 1 \| \|  \| Unidentified sp. M \| 0 \| 1 \| \|  \| Unidentified sp. N \| 0 \| 1 \| \|  \| Unidentified sp. O \| 0 \| 1 \| \|  \| Unidentified sp. P \| 1 \| 0 \| \|  \| Unidentified sp. Q \| 1 \| 0 \| |
| --- | --- | --- | --- | --- | --- | --- | --- | --- | --- | --- | --- | --- | --- | --- | --- | --- | --- | --- | --- | --- | --- | --- | --- | --- | --- | --- | --- | --- | --- | --- | --- | --- | --- | --- | --- | --- | --- | --- | --- | --- | --- | --- | --- | --- | --- | --- | --- | --- | --- | --- | --- | --- | --- | --- | --- | --- | --- | --- | --- | --- | --- | --- | --- | --- | --- | --- | --- | --- | --- | --- | --- | --- | --- | --- | --- | --- | --- | --- | --- | --- | --- | --- | --- | --- | --- | --- | --- | --- | --- | --- | --- | --- | --- | --- | --- | --- | --- | --- | --- | --- | --- | --- | --- | --- | --- | --- | --- | --- | --- | --- | --- | --- | --- | --- | --- | --- | --- | --- | --- | --- | --- | --- | --- | --- | --- | --- | --- | --- | --- | --- | --- | --- | --- | --- | --- | --- | --- | --- | --- | --- | --- | --- | --- | --- | --- | --- | --- | --- | --- | --- | --- | --- | --- | --- | --- | --- | --- | --- | --- | --- | --- | --- | --- | --- | --- | --- | --- | --- | --- | --- | --- | --- | --- | --- | --- | --- | --- | --- | --- | --- | --- | --- | --- | --- | --- | --- | --- | --- | --- | --- | --- | --- | --- | --- | --- | --- | --- | --- | --- | --- | --- | --- | --- | --- | --- | --- | --- | --- | --- | --- | --- | --- | --- | --- | --- | --- | --- | --- | --- | --- | --- | --- | --- | --- | --- | --- | --- | --- | --- | --- | --- | --- | --- | --- | --- | --- | --- | --- | --- | --- | --- | --- | --- | --- | --- | --- | --- | --- | --- | --- | --- | --- | --- | --- | --- | --- | --- | --- | --- | --- | --- | --- | --- | --- | --- | --- | --- | --- | --- | --- | --- | --- | --- | --- | --- | --- | --- | --- | --- | --- | --- | --- | --- | --- | --- | --- | --- | --- | --- | --- | --- | --- | --- | --- | --- | --- | --- | --- | --- | --- | --- | --- | --- | --- | --- | --- | --- | --- | --- | --- | --- | --- | --- | --- | --- | --- | --- | --- | --- | --- | --- | --- | --- | --- | --- | --- | --- | --- | --- | --- | --- | --- | --- | --- | --- | --- | --- | --- | --- | --- | --- | --- | --- | --- | --- | --- | --- | --- | --- | --- | --- | --- | --- | --- | --- | --- | --- | --- | --- | --- | --- | --- | --- | --- | --- | --- | --- | --- | --- | --- | --- | --- | --- | --- | --- | --- | --- | --- | --- | --- | --- | --- | --- | --- | --- | --- | --- | --- | --- | --- | --- | --- | --- | --- | --- | --- | --- | --- | --- | --- | --- | --- | --- | --- | --- | --- | --- | --- | --- | --- | --- | --- | --- | --- | --- | --- | --- | --- | --- | --- | --- | --- | --- | --- | --- | --- | --- | --- | --- | --- | --- | --- | --- | --- | --- | --- | --- | --- | --- | --- | --- | --- | --- | --- | --- | --- | --- | --- | --- | --- | --- | --- | --- | --- | --- | --- | --- | --- | --- | --- | --- | --- | --- | --- | --- | --- | --- | --- | --- | --- | --- | --- | --- | --- | --- | --- | --- | --- | --- | --- | --- | --- | --- | --- | --- | --- | --- | --- | --- | --- | --- | --- | --- | --- | --- | --- | --- | --- | --- | --- | --- | --- | --- | --- | --- | --- | --- | --- | --- | --- | --- | --- | --- | --- | --- | --- | --- | --- | --- | --- | --- | --- | --- | --- | --- | --- | --- | --- | --- | --- | --- | --- | --- | --- | --- | --- | --- | --- | --- | --- | --- | --- | --- | --- | --- | --- | --- | --- | --- | --- | --- | --- | --- | --- | --- | --- | --- | --- | --- | --- | --- | --- | --- | --- | --- | --- | --- | --- | --- | --- | --- | --- | --- | --- | --- | --- | --- | --- | --- | --- | --- | --- | --- | --- | --- | --- | --- | --- | --- | --- | --- | --- | --- | --- | --- | --- | --- | --- | --- | --- | --- | --- | --- | --- | --- | --- | --- | --- | --- | --- | --- | --- | --- | --- | --- | --- | --- | --- | --- | --- | --- | --- | --- | --- | --- | --- | --- | --- | --- | --- | --- | --- | --- | --- | --- | --- | --- | --- | --- | --- | --- | --- | --- | --- | --- | --- | --- | --- | --- | --- | --- | --- | --- | --- | --- | --- | --- | --- | --- | --- | --- | --- | --- | --- | --- | --- | --- | --- | --- | --- | --- | --- | --- | --- | --- | --- | --- | --- | --- | --- | --- | --- | --- | --- | --- | --- | --- | --- | --- | --- | --- | --- | --- | --- | --- | --- | --- | --- | --- | --- | --- | --- | --- | --- | --- | --- | --- | --- | --- | --- | --- | --- | --- | --- | --- | --- | --- | --- | --- | --- | --- | --- | --- | --- | --- | --- | --- | --- | --- | --- | --- | --- | --- | --- | --- | --- | --- | --- | --- | --- | --- | --- | --- | --- | --- | --- | --- | --- | --- | --- | --- | --- | --- | --- | --- | --- | --- | --- | --- | --- | --- | --- | --- | --- | --- | --- | --- | --- | --- | --- | --- | --- | --- | --- | --- | --- | --- | --- | --- | --- | --- | --- | --- | --- | --- | --- | --- | --- | --- | --- | --- | --- | --- | --- | --- | --- | --- | --- | --- | --- | --- | --- | --- | --- | --- | --- | --- | --- | --- | --- | --- | --- | --- | --- | --- | --- | --- | --- | --- | --- | --- | --- | --- | --- | --- | --- | --- | --- | --- | --- | --- | --- | --- | --- | --- | --- | --- | --- | --- | --- | --- | --- | --- | --- | --- | --- | --- | --- | --- | --- | --- | --- |
