# Supplementary material for: Mixed-Forest Species Establishment in a Monodominant Forest in Central Africa: Implications for Tropical Forest Invasibility
Source: PLoS One. 2014 May 20;9(5):e97585. doi: 10.1371/journal.pone.0097585 (PMC4028239; doi:10.1371/journal.pone.0097585)
Supplement: Table S2 — Traits and life histories of all tree species. Life-history trait data set for the 193 species at the Dja Faunal Reserve based on published literature (e.g., Lewis et al. 2009; Sonké 2004; Poorter et al. 2003; van Gemerden et al. 2003), herbarium specimens and personal observations. The relative abundance of each species in the mixed forests was based on the three mixed forest plots. Wood mass density is defined as dry wood mass/green wood volume (g cm−3) and is compiled from Lewis et al. (2009). Species were classified according to maximum stature in three classes: large trees (>30 m tall), medium trees (10–30 m tall) and small trees (<10 m tall). Species were placed on the basis of maximum dbh in three classes: large diameter (>100 cm in dbh), medium diameter (50 cm–100 cm) and small diameter (<50 cm). Each species is classified in one of two categories according to its fruit/seed dispersal mode (biotic and non-biotic dependent). Each species was classified into one of two categories according to its ecological guild in terms of light requirement (pioneer [e.g., light-demanders that require high light level for seedling establishment], and non-pioneer [shade-bearers that are capable of seedling establishment under forest shade, though some shade-bears may need higher light level at later stage of life]), and each was grouped according to its geographical distribution (narrow, i.e., species endemic to lower-Guinea-Congolean biogeographical region; and wide that includes species which were not endemic to the region). (DOCX) [file pone.0097585.s002.docx]

| \|  \|  \|  \|  \|  \|  \|  \|  \|  \|  \|  \|  \| \| --- \| --- \| --- \| --- \| --- \| --- \| --- \| --- \| --- \| --- \| --- \| --- \| \|  \| Species name \| Family \| Presence \| Relative \| Wood mass \| Maximum \| Diameter at \| Dispersal \| Light \| Geographical \|  \| \|  \|  \|  \|  \| abundance \| density (g cm^-3^) \| height \| breast height \| mechanism \| requirement \| distribution \|  \| \|  \| *Allanblackia floribunda* \| Clusiaceae \| 0 \| 4 \| 0.723 \| large \| medium \| biotic \| pioneer \| narrow \|  \| \|  \| *Alstonia boonei* \| Apocynaceae \| 1 \| 7 \| 0.323 \| large \| large \| non-biotic \| pioneer \| narrow \|  \| \|  \| *Amphirmas pterocarpoides* \| Caesalpiniaceae \| 0 \| 4 \| 0.565 \| large \| large \| non-biotic \| non-pioneer \| narrow \|  \| \|  \| *Angylocalyx pynaerthii* \| Fabaceae \| 1 \| 6 \| 0.655 \| small \| small \| biotic \| non-pioneer \| narrow \|  \| \|  \| *Anonidium mannii* \| Annonaceae \| 1 \| 95 \| 0.293 \| medium \| medium \| biotic \| non-pioneer \| narrow \|  \| \|  \| *Anopyxis klaineana* \| Anisophylleaceae \| 1 \| 0 \| 0.811 \| large \| large \| biotic \| non-pioneer \| narrow \|  \| \|  \| *Anthonotha cladantha* \| Caesalpiniaceae \| 1 \| 1 \| 0.577 \| medium \| medium \| non-biotic \| non-pioneer \| narrow \|  \| \|  \| *Anthonotha macrophylla* \| Caesalpiniaceae \| 0 \| 9 \| 0.849 \| medium \| small \| non-biotic \| non-pioneer \| narrow \|  \| \|  \| *Antidesma sp. A* \| Eurphorbiaceae \| 0 \| 1 \| 0.613 \|  \|  \|  \|  \|  \|  \| \|  \| *Baillonella toxisperma* \| Sapotaceae \| 1 \| 0 \| 0.729 \| large \| large \| biotic \| non-pioneer \| narrow \|  \| \|  \| *Beilschmiedia sp. A* \| Lauraceae \| 0 \| 1 \| 0.637 \|  \|  \|  \|  \|  \|  \| \|  \| *Beilschmiedia sp. B* \| Lauraceae \| 0 \| 1 \| 0.637 \|  \|  \|  \|  \|  \|  \| \|  \| *Blighia sapida* \| Sapindaceae \| 1 \| 12 \| 0.784 \| medium \| medium \| biotic \| non-pioneer \| narrow \|  \| \|  \| *Blighia welwitschii* \| Sapindaceae \| 1 \| 4 \| 0.820 \| large \| large \| biotic \| non-pioneer \| narrow \|  \| \|  \| *Brenania brieyi* \| Rubiaceae \| 0 \| 2 \| 0.541 \| large \| medium \| biotic \| non-pioneer \| narrow \|  \| \|  \| *Calpocalyx dinklagei* \| Mimosaceae \| 0 \| 1 \| 0.648 \| small \| small \| biotic \| non-pioneer \| narrow \|  \| \|  \| *Canarium schweinfurthll* \| Burseraceae \| 0 \| 1 \| 0.414 \| large \| large \| biotic \| non-pioneer \| narrow \|  \| \|  \| *Canthium sp. A* \| Rubiaceae \| 0 \| 1 \| 0.661 \|  \|  \|  \|  \|  \|  \| \|  \| *Carapa procera* \| Melicaceae \| 1 \| 66 \| 0.630 \| large \| large \| biotic \| non-pioneer \| wide \|  \| \|  \| *Carapa sp. A* \| Melicaceae \| 0 \| 4 \| 0.555 \|  \|  \|  \|  \|  \|  \| \|  \| *Ceiba pentandra* \| Bombaceae \| 0 \| 1 \| 0.277 \| large \| large \| non-biotic \| pioneer \| wide \|  \| \|  \| *Celtis tessmannii* \| Ulmaceae \| 1 \| 15 \| 0.656 \| large \| large \| biotic \| non-pioneer \| narrow \|  \| \|  \| *Celtis zenkeri* \| Ulmaceae \| 1 \| 23 \| 0.620 \| large \| large \| biotic \| non-pioneer \| narrow \|  \| \|  \| *Centroplacus glaucinus* \| Eurphorbiaceae \| 1 \| 24 \| 0.523 \| small \| small \| biotic \| non-pioneer \| narrow \|  \| \|  \| *Chytranthus* sp. A \| Sapindaceae \| 0 \| 1 \| 0.593 \|  \|  \|  \|  \|  \|  \| \|  \| *Chytranthus* sp. B \| Sapindaceae \| 0 \| 1 \| 0.593 \|  \|  \|  \|  \|  \|  \| \|  \| *Chytranthus* sp. C \| Sapindaceae \| 0 \| 1 \| 0.593 \|  \|  \|  \|  \|  \|  \| \|  \| *Chytranthus* sp. D \| Sapindaceae \| 0 \| 1 \| 0.593 \|  \|  \|  \|  \|  \|  \| \|  \| *Chytranthus* sp. E \| Sapindaceae \| 0 \| 1 \| 0.593 \|  \|  \|  \|  \|  \|  \| \|  \| *Chytranthus* sp. F \| Sapindaceae \| 0 \| 1 \| 0.593 \|  \|  \|  \|  \|  \|  \| \|  \| *Cleistopholis glauca* \| Annonaceae \| 0 \| 5 \| 0.309 \| medium \| small \| biotic \| non-pioneer \| narrow \|  \| \|  \| *Cleistopholis patens* \| Annonaceae \| 0 \| 1 \| 0.348 \| medium \| medium \| biotic \| non-pioneer \| narrow \|  \| \|  \| *Coelocaryon preussii* \| Myristicaceae \| 0 \| 6 \| 0.499 \| medium \| medium \| biotic \| non-pioneer \| narrow \|  \| \|  \| *Coffea* sp. A \| Rubiaceae \| 0 \| 1 \| 0.541 \|  \|  \|  \|  \|  \|  \| \|  \| *Cola acuminata* \| Sterculiaceae \| 1 \| 6 \| 0.556 \| medium \| small \| biotic \| non-pioneer \| narrow \|  \| \|  \| *Cola lateritia* \| Sterculiaceae \| 0 \| 8 \| 0.590 \| large \| medium \| biotic \| non-pioneer \| narrow \|  \| \|  \| *Cylicodiscus gabonensis* \| Mimosaceae \| 0 \| 2 \| 0.799 \| large \| large \| non-biotic \| non-pioneer \| narrow \|  \| \|  \| *Dacryodes edulis* \| Burseraceae \| 0 \| 7 \| 0.528 \| medium \| medium \| biotic \| pioneer \| narrow \|  \| \|  \| *Dacryodes* sp. A \| Burseraceae \| 0 \| 1 \| 0.554 \|  \|  \|  \|  \|  \|  \| \|  \| *Desbordesia glaucescens* \| Irvingiaceae \| 1 \| 13 \| 0.921 \| large \| large \| non-biotic \| non-pioneer \| narrow \|  \| \|  \| *Desplatsia chrysochlamys* \| Tiliaceae \| 0 \| 4 \| 0.561 \| small \| small \| biotic \| non-pioneer \| narrow \|  \| \|  \| *Desplatsia dewevrei* \| Tiliaceae \| 0 \| 11 \| 0.561 \| small \| small \| biotic \| non-pioneer \| narrow \|  \| \|  \| *Dialium guineensis* \| Caesalpiniaceae \| 1 \| 5 \| 0.885 \| medium \| medium \| non-biotic \| non-pioneer \| narrow \|  \| \|  \| *Diospyros crassifolia* \| Ebenaceae \| 1 \| 4 \| 0.881 \| medium \| large \| biotic \| non-pioneer \| narrow \|  \| \|  \| *Diospyros hoyleana* \| Ebenaceae \| 0 \| 5 \| 0.826 \| small \| small \| biotic \| non-pioneer \| narrow \|  \| \|  \| *Discoglypremna caloneura* \| Eurphorbiaceae \| 0 \| 3 \| 0.351 \| medium \| medium \| biotic \| pioneer \| narrow \|  \| \|  \| *Distemonanthus benthamianus* \| Caesalpiniaceae \| 0 \| 31 \| 0.575 \| large \| large \| non-biotic \| non-pioneer \| narrow \|  \| \|  \| *Donella pruniformis* \| Sapotaceae \| 0 \| 2 \| 0.635 \| large \| medium \| biotic \| non-pioneer \| narrow \|  \| \|  \| *Drypetes cf similis* \| Eurphorbiaceae \| 0 \| 2 \| 0.671 \|  \|  \|  \|  \|  \|  \| \|  \| *Drypetes chevalieleri* \| Eurphorbiaceae \| 1 \| 3 \| 0.630 \| small \| small \| biotic \| non-pioneer \| narrow \|  \| \|  \| *Drypetes floribunda* \| Eurphorbiaceae \| 0 \| 2 \| 0.671 \| small \|  \|  \|  \|  \|  \| \|  \| *Drypetes goosweleiri* \| Eurphorbiaceae \| 1 \| 0 \| 0.672 \| large \| small \| biotic \| non-pioneer \| narrow \|  \| \|  \| *Drypetes ivorensis* \| Eurphorbiaceae \| 1 \| 1 \| 0.671 \| large \| small \| biotic \| non-pioneer \| narrow \|  \| \|  \| *Drypetes laciniata* \| Eurphorbiaceae \| 0 \| 3 \| 0.630 \| small \| small \| biotic \| non-pioneer \| narrow \|  \| \|  \| *Drypetes* sp. A \| Eurphorbiaceae \| 0 \| 1 \| 0.671 \|  \|  \|  \|  \|  \|  \| \|  \| *Drypetes* sp. B \| Eurphorbiaceae \| 0 \| 1 \| 0.671 \|  \|  \|  \|  \|  \|  \| \|  \| *Drypetes* sp. C \| Eurphorbiaceae \| 0 \| 1 \| 0.671 \|  \|  \|  \|  \|  \|  \| \|  \| *Drypetes* sp. D \| Eurphorbiaceae \| 0 \| 1 \| 0.671 \|  \|  \|  \|  \|  \|  \| \|  \| *Drypetes* sp. E \| Eurphorbiaceae \| 0 \| 1 \| 0.671 \|  \|  \|  \|  \|  \|  \| \|  \| *Drypetes* sp. F \| Eurphorbiaceae \| 0 \| 1 \| 0.671 \|  \|  \|  \|  \|  \|  \| \|  \| *Duboscia macrocarpa* \| Tiliaceae \| 1 \| 3 \| 0.561 \| large \| large \| biotic \| non-pioneer \| narrow \|  \| \|  \| *Enantia chlorantha* \| Annonaceae \| 1 \| 24 \| 0.424 \| medium \| small \| biotic \| non-pioneer \| narrow \|  \| \|  \| *Entandrophragma angolense* \| Melicaceae \| 0 \| 1 \| 0.497 \| large \| large \| non-biotic \| non-pioneer \| narrow \|  \| \|  \| *Entandrophragma cylindricum* \| Melicaceae \| 1 \| 1 \| 0.577 \| large \| large \| non-biotic \| non-pioneer \| narrow \|  \| \|  \| *Eribroma oblongum* \| Sterculiaceae \| 0 \| 1 \| 0.590 \| large \| large \| biotic \| non-pioneer \| narrow \|  \| \|  \| *Eriocoelum macrocarpum* \| Sapindaceae \| 1 \| 23 \| 0.500 \| medium \| medium \| non-biotic \| non-pioneer \| narrow \|  \| \|  \| *Erismadelphus exul* \| Vochysiaceae \| 0 \| 1 \| 0.608 \| large \| large \| non-biotic \| non-pioneer \| narrow \|  \| \|  \| *Erythrophloeum suaveolens* \| Caesalpiniaceae \| 1 \| 4 \| 0.842 \| large \| large \| non-biotic \| non-pioneer \| wide \|  \| \|  \| *Eugenia* sp. B \| Myrtaceae \| 0 \| 1 \| 0.613 \|  \|  \|  \|  \|  \|  \| \|  \| *Eugenia* sp. A \| Myrtaceae \| 0 \| 1 \| 0.613 \|  \|  \|  \|  \|  \|  \| \|  \| *Fernandoa adolphi-fredirici* \| Bigononiaceae \| 1 \| 1 \| 0.589 \| medium \| medium \| non-biotic \| pioneer \| narrow \|  \| \|  \| *Ficus* sp. A \| Moraceae \| 0 \| 1 \| 0.540 \|  \|  \|  \|  \|  \|  \| \|  \| *Funtumia africana* \| Apocynaceae \| 0 \| 1 \| 0.449 \| medium \| small \| non-biotic \| pioneer \| narrow \|  \| \|  \| *Funtumia elastica* \| Apocynaceae \| 0 \| 5 \| 0.425 \| medium \| small \| non-biotic \| pioneer \| narrow \|  \| \|  \| *Gambeya lacourtiana* \| Sapotaceae \| 1 \| 10 \| 0.611 \| large \| large \| biotic \| non-pioneer \| narrow \|  \| \|  \| *Gambeya perpulchra* \| Sapotaceae \| 0 \| 1 \| 0.595 \|  \|  \|  \|  \|  \|  \| \|  \| *Gambeya* sp. A \| Sapotaceae \| 0 \| 1 \| 0.595 \|  \|  \|  \|  \|  \|  \| \|  \| *Gambeya* sp. B \| Sapotaceae \| 0 \| 1 \| 0.595 \|  \|  \|  \|  \|  \|  \| \|  \| *Garcinia mannii* \| Clusiaceae \| 0 \| 10 \| 0.835 \| medium \| small \| biotic \| non-pioneer \| narrow \|  \| \|  \| *Garcinia punctata* \| Clusiaceae \| 0 \| 1 \| 0.835 \| large \| small \| biotic \| non-pioneer \| narrow \|  \| \|  \| *Guarea cedrata* \| Melicaceae \| 1 \| 8 \| 0.516 \| large \| large \| biotic \| non-pioneer \| narrow \|  \| \|  \| *Guarea thompsonii* \| Melicaceae \| 1 \| 14 \| 0.563 \| large \| large \| biotic \| non-pioneer \| narrow \|  \| \|  \| *Heisteria trillesiana* \| Olacaeae \| 1 \| 18 \| 0.734 \| medium \| large \| biotic \| non-pioneer \| narrow \|  \| \|  \| *Heisteria zimmereri* \| Olacaeae \| 0 \| 1 \| 0.734 \| large \| large \| biotic \| non-pioneer \| narrow \|  \| \|  \| *Hexalobus crispiliformis* \| Annonaceae \| 0 \| 3 \| 0.486 \| medium \| large \| biotic \| non-pioneer \| narrow \|  \| \|  \| *Homalium dolichophyllum* \| Flacourtiaceae \| 0 \| 1 \| 0.737 \| medium \| large \| biotic \| non-pioneer \| narrow \|  \| \|  \| *Homalium letestui* \| Flacourtiaceae \| 0 \| 1 \| 0.725 \| medium \| small \| biotic \| non-pioneer \| narrow \|  \| \|  \| *Hylodendron gabunense* \| Caesalpiniaceae \| 1 \| 6 \| 0.792 \| medium \| large \| non-biotic \| non-pioneer \| narrow \|  \| \|  \| *Hymenocardia lyrata* \| Eurphorbiaceae \| 0 \| 4 \| 0.523 \| medium \| medium \| non-biotic \| pioneer \| narrow \|  \| \|  \| *Hymenocardia* sp. A \| Eurphorbiaceae \| 0 \| 2 \| 0.523 \|  \|  \|  \|  \|  \|  \| \|  \| *Irvingia gabonensis* \| Irvingiaceae \| 1 \| 5 \| 0.794 \| medium \| large \| biotic \| non-pioneer \| narrow \|  \| \|  \| *Irvingia grandifolia* \| Irvingiaceae \| 1 \| 2 \| 0.803 \| large \| large \| biotic \| non-pioneer \| narrow \|  \| \|  \| *Irvingia robur* \| Irvingiaceae \| 1 \| 1 \| 0.707 \| large \| large \| biotic \| non-pioneer \| narrow \|  \| \|  \| *Isomacrolobium* sp. A \| Fabaceae \| 0 \| 3 \| 0.655 \|  \|  \|  \|  \|  \|  \| \|  \| *Keayodendron bridelioides* \| Eurphorbiaceae \| 0 \| 4 \| 0.523 \| medium \| medium \| biotic \| non-pioneer \| narrow \|  \| \|  \| *Klaineanthus gaboniae* \| Eurphorbiaceae \| 0 \| 3 \| 0.523 \| medium \| small \| biotic \| non-pioneer \| narrow \|  \| \|  \| *Klaineanthus* sp. A \| Eurphorbiaceae \| 0 \| 1 \| 0.523 \|  \|  \|  \|  \|  \|  \| \|  \| *Klainedoxa gabonensis* \| Irvingiaceae \| 1 \| 7 \| 0.940 \| large \| large \| biotic \| non-pioneer \| narrow \|  \| \|  \| *Lannea welwitschii* \| Anacardiaceae \| 0 \| 1 \| 0.425 \| large \| large \| biotic \| non-pioneer \| narrow \|  \| \|  \| *Lasiodiscus mannii* \| Rhamnaceae \| 1 \| 0 \| 0.379 \| small \| small \| biotic \| non-pioneer \| narrow \|  \| \|  \| *Lepidobotrys staudtii* \| Lepidobotryaceae \| 1 \| 12 \|  \| medium \| medium \| biotic \| non-pioneer \| narrow \|  \| \|  \| *Licania elaeosperma* \| Chrysobalanaceae \| 0 \| 1 \| 0.702 \|  \|  \|  \|  \|  \|  \| \|  \| *Lovoa trichiloides* \| Melicaceae \| 1 \| 3 \| 0.460 \| large \| large \| non-biotic \| non-pioneer \| narrow \|  \| \|  \| *Macaranga grandis* \| Eurphorbiaceae \| 0 \| 3 \| 0.394 \| medium \| small \| biotic \| pioneer \| narrow \|  \| \|  \| *Macaranga spinosa* \| Eurphorbiaceae \| 0 \| 4 \| 0.394 \| medium \| small \| biotic \| pioneer \| narrow \|  \| \|  \| *Maesobotrya dusenii* \| Eurphorbiaceae \| 0 \| 6 \| 0.525 \|  \|  \|  \|  \|  \|  \| \|  \| *Maesopsis eminii* \| Rhamnaceae \| 0 \| 2 \| 0.396 \| large \| medium \| biotic \| pioneer \| narrow \|  \| \|  \| *Magnistipula* sp. A \| Clusiaceae \| 0 \| 1 \| 0.702 \|  \|  \|  \|  \|  \|  \| \|  \| *Mammea africana* \| Clusiaceae \| 1 \| 1 \| 0.633 \| large \| large \| biotic \| non-pioneer \| narrow \|  \| \|  \| *Manilkara letouzeyi* \| Sapotaceae \| 0 \| 1 \| 0.828 \| large \| large \| biotic \| non-pioneer \| narrow \|  \| \|  \| *Maranthes glabra* \| Chrysobalanaceae \| 0 \| 2 \| 0.881 \| large \| large \| biotic \| non-pioneer \| narrow \|  \| \|  \| *Maranthes* sp. A \| Chrysobalanaceae \| 0 \| 1 \| 0.702 \|  \|  \|  \|  \|  \|  \| \|  \| *Mareyopsis longifolia* \| Eurphorbiaceae \| 1 \| 0 \| 0.523 \| small \| small \| biotic \| non-pioneer \| narrow \|  \| \|  \| *Margaritaria discoidea* \| Eurphorbiaceae \| 0 \| 3 \| 0.776 \| large \| large \| biotic \| pioneer \| wide \|  \| \|  \| *Memecylon amshoffiae* \| Melastomataceae \| 0 \| 1 \| 0.824 \| small \| small \|  \|  \|  \|  \| \|  \| *Microdesmis puberula* \| Pandaceae \| 0 \| 2 \|  \| small \| small \| biotic \| non-pioneer \| narrow \|  \| \|  \| *Milicia exelsa* \| Moraceae \| 0 \| 1 \| 0.575 \| large \| large \| biotic \| pioneer \| narrow \|  \| \|  \| *Millettia laurentii* \| Fabaceae \| 0 \| 1 \| 0.764 \| medium \| small \| non-biotic \| non-pioneer \| narrow \|  \| \|  \| *Musanga cecropioides* \| Moraceae \| 0 \| 1 \| 0.247 \| medium \| large \| biotic \| pioneer \| narrow \|  \| \|  \| *Myrianthus arboreus* \| Moraceae \| 0 \| 21 \| 0.454 \| medium \| small \| biotic \| pioneer \| narrow \|  \| \|  \| *Nauclea diderrichii* \| Rubiaceae \| 0 \| 3 \| 0.673 \| large \| medium \| biotic \| pioneer \| narrow \|  \| \|  \| *Odyendya gabonensis* \| Simaroubaceae \| 1 \| 2 \| 0.327 \| medium \| large \| biotic \| non-pioneer \| narrow \|  \| \|  \| *Oncoba glauca* \| Flacourtiaceae \| 0 \| 29 \| 0.652 \| medium \| small \|  \|  \|  \|  \| \|  \| *Ongokea gore* \| Olacaeae \| 1 \| 2 \| 0.769 \| large \| large \| biotic \| non-pioneer \| narrow \|  \| \|  \| *Pachypodanthium staudtii* \| Annonaceae \| 0 \| 3 \| 0.580 \| medium \| medium \| biotic \| non-pioneer \| narrow \|  \| \|  \| *Pancovia pedicellaris* \| Sapindaceae \| 0 \| 1 \| 0.635 \| medium \| small \| biotic \| non-pioneer \| narrow \|  \| \|  \| *Panda oleosa* \| Pandaceae \| 1 \| 15 \|  \| large \| medium \| biotic \| non-pioneer \| narrow \|  \| \|  \| *Parkia bicolor* \| Mimosaceae \| 0 \| 5 \| 0.469 \| large \| large \| biotic \| non-pioneer \| narrow \|  \| \|  \| *Pauridiantha floribunda* \| Rubiaceae \| 0 \| 2 \| 0.541 \| small \| small \| biotic \| pioneer \| narrow \|  \| \|  \| *Pausynistalia macroceras* \| Rubiaceae \| 0 \| 1 \| 0.560 \| medium \| small \| biotic \| non-pioneer \| narrow \|  \| \|  \| *Pentaclethra macrophylla* \| Mimosaceae \| 1 \| 45 \| 0.867 \| medium \| large \| non-biotic \| non-pioneer \| narrow \|  \| \|  \| *Petersianthus macrocarpus* \| Lecythidaceae \| 1 \| 60 \| 0.690 \| large \| large \| non-biotic \| non-pioneer \| narrow \|  \| \|  \| *Picralima nitida* \| Apocynaceae \| 0 \| 3 \| 0.785 \| medium \| small \| biotic \| non-pioneer \| narrow \|  \| \|  \| *Piptadeniastrum africana* \| Mimosaceae \| 0 \| 6 \| 0.613 \| large \| large \| non-biotic \| non-pioneer \| narrow \|  \| \|  \| *Plagiostyles africana* \| Eurphorbiaceae \| 1 \| 0 \| 0.748 \| medium \| small \| biotic \| non-pioneer \| narrow \|  \| \|  \| *Polyalthia suaveolens* \| Annonaceae \| 1 \| 67 \| 0.704 \| medium \| small \| biotic \| non-pioneer \| narrow \|  \| \|  \| *Pseudospondias microcarpa* \| Anacardiaceae \| 0 \| 4 \| 0.461 \| medium \| medium \| biotic \| non-pioneer \| wide \|  \| \|  \| *Pteleopsis hylodendron* \| Combretaceae \| 0 \| 1 \| 0.681 \| large \| large \| non-biotic \| non-pioneer \| narrow \|  \| \|  \| *Pterocarpus mildbreadii* \| Fabaceae \| 0 \| 4 \| 0.589 \| large \| large \| non-biotic \| non-pioneer \| narrow \|  \| \|  \| *Pterocarpus soyauxii* \| Fabaceae \| 1 \| 11 \| 0.661 \| large \| large \| non-biotic \| non-pioneer \| narrow \|  \| \|  \| *Pycnanthus angolensis* \| Myristicaceae \| 0 \| 5 \| 0.414 \| large \| large \| biotic \| pioneer \| narrow \|  \| \|  \| *Rauvolfia vomitoria* \| Apocynaceae \| 0 \| 3 \| 0.484 \| small \| small \| biotic \| pioneer \| narrow \|  \| \|  \| *Rhabdophyllum* sp. A \| Ochnaceae \| 0 \| 1 \| 0.752 \|  \|  \|  \|  \|  \|  \| \|  \| *Rhabdophyllum* sp. B \| Ochnaceae \| 0 \| 1 \| 0.752 \|  \|  \|  \|  \|  \|  \| \|  \| *Rhabdophyllum* sp. C \| Ochnaceae \| 0 \| 2 \| 0.752 \|  \|  \|  \|  \|  \|  \| \|  \| *Rhabdophyllum* sp. D \| Ochnaceae \| 1 \| 0 \| 0.752 \|  \|  \|  \|  \|  \|  \| \|  \| *Rinorea oblongifolia* \| Violaceae \| 0 \| 3 \|  \| small \| small \| biotic \| non-pioneer \| narrow \|  \| \|  \| *Rinorea* sp. A \| Violaceae \| 1 \| 0 \|  \|  \|  \|  \|  \|  \|  \| \|  \| *Rinorea welwitschii* \| Violaceae \| 0 \| 1 \|  \| small \| small \| biotic \| non-pioneer \| narrow \|  \| \|  \| *Rothmannia lujae* \| Rubiaceae \| 1 \| 20 \| 0.541 \| medium \| small \| biotic \| non-pioneer \| narrow \|  \| \|  \| *Santiria trimera* \| Burseraceae \| 1 \| 30 \| 0.549 \| medium \| medium \| biotic \| non-pioneer \| narrow \|  \| \|  \| *Scottellia* sp. A \| Flacourtiaceae \| 0 \| 1 \| 0.549 \|  \|  \|  \|  \|  \|  \| \|  \| *Sorindeia grandifolia* \| Anacardiaceae \| 1 \| 18 \| 0.560 \| medium \| small \| biotic \| non-pioneer \| narrow \|  \| \|  \| *Sorindeia mildbraedii* \| Anacardiaceae \| 1 \| 12 \| 0.560 \| medium \| small \| biotic \| non-pioneer \| narrow \|  \| \|  \| *Staudtia stipitata* \| Myristicaceae \| 1 \| 8 \| 0.723 \| medium \| large \| biotic \| non-pioneer \| narrow \|  \| \|  \| *Sterculia tragacantha* \| Sterculiaceae \| 0 \| 1 \| 0.641 \| medium \| medium \| biotic \| pioneer \| wide \|  \| \|  \| *Strombosia grandifolia* \| Olacaeae \| 1 \| 5 \| 0.843 \| medium \| small \| biotic \| non-pioneer \| narrow \|  \| \|  \| *Strombosia pustulata* \| Olacaeae \| 1 \| 3 \| 0.854 \| medium \| small \| biotic \| non-pioneer \| narrow \|  \| \|  \| *Strombosia zenkeri* \| Olacaeae \| 0 \| 1 \| 0.777 \| medium \| medium \| biotic \| non-pioneer \| narrow \|  \| \|  \| *Strombosiopsis tetandra* \| Olacaeae \| 1 \| 8 \| 0.671 \| medium \| large \| biotic \| non-pioneer \| narrow \|  \| \|  \| *Symphonia globulifera* \| Clusiaceae \| 0 \| 2 \| 0.601 \| large \| large \| biotic \| non-pioneer \| wide \|  \| \|  \| *Synsepalum dulcificum* \| Sapotaceae \| 1 \| 11 \| 0.678 \| medium \| small \| biotic \| non-pioneer \| narrow \|  \| \|  \| *Syzygium rowlandii* \| Myrtaceae \| 0 \| 4 \| 0.611 \| medium \| medium \| biotic \| non-pioneer \| narrow \|  \| \|  \| *Tabernaemontana crassa* \| Apocynaceae \| 1 \| 54 \| 0.640 \| medium \| small \| biotic \| pioneer \| narrow \|  \| \|  \| *Tessmannia africana* \| Caesalpiniaceae \| 1 \| 1 \| 0.835 \| large \| large \| non-biotic \| non-pioneer \| narrow \|  \| \|  \| *Tessmannia anomala* \| Caesalpiniaceae \| 1 \| 0 \| 0.797 \|  \|  \|  \|  \|  \|  \| \|  \| *Tetrapleura tetraptera* \| Mimosaceae \| 0 \| 3 \| 0.588 \| medium \| medium \| non-biotic \| non-pioneer \| narrow \|  \| \|  \| *Treculia africana* \| Moraceae \| 1 \| 1 \| 0.454 \| medium \| medium \| biotic \| non-pioneer \| wide \|  \| \|  \| *Tricalysia discolor* \| Rubiaceae \| 0 \| 2 \| 0.541 \| medium \| small \| biotic \| non-pioneer \| narrow \|  \| \|  \| *Tricalysia* sp. A \| Rubiaceae \| 0 \| 1 \| 0.541 \|  \|  \|  \|  \|  \|  \| \|  \| *Tricalysia* sp. B \| Rubiaceae \| 0 \| 1 \| 0.541 \|  \|  \|  \|  \|  \|  \| \|  \| *Trichilia prieuriana* \| Melicaceae \| 0 \| 1 \| 0.671 \| medium \| small \| biotic \| non-pioneer \| narrow \|  \| \|  \| *Trichilia rubescens* \| Melicaceae \| 0 \| 12 \| 0.565 \| medium \| large \| biotic \| non-pioneer \| narrow \|  \| \|  \| *Trichilia welwitschii* \| Melicaceae \| 1 \| 3 \| 0.565 \| medium \| medium \| biotic \| non-pioneer \| narrow \|  \| \|  \| *Trichoscypha acuminata* \| Anacardiaceae \| 1 \| 22 \| 0.615 \| small \| small \| biotic \| non-pioneer \| narrow \|  \| \|  \| *Trichoscypha arborea* \| Anacardiaceae \| 0 \| 1 \| 0.670 \| large \| large \| biotic \| non-pioneer \| narrow \|  \| \|  \| *Tridesmostemon omphalocarpoides* \| Sapotaceae \| 0 \| 2 \| 0.635 \| large \| large \| biotic \| non-pioneer \| narrow \|  \| \|  \| *Trilepisium madascariensis* \| Moraceae \| 0 \| 1 \| 0.454 \| medium \| medium \| biotic \| non-pioneer \| narrow \|  \| \|  \| *Uapaca acuminata* \| Eurphorbiaceae \| 1 \| 20 \| 0.613 \| medium \| large \| biotic \| non-pioneer \| narrow \|  \| \|  \| *Uapaca guineensis* \| Eurphorbiaceae \| 0 \| 23 \| 0.642 \| medium \| medium \| biotic \| non-pioneer \| narrow \|  \| \|  \| *Uapaca paluosa* \| Eurphorbiaceae \| 0 \| 34 \| 0.705 \| large \| large \| biotic \| non-pioneer \| narrow \|  \| \|  \| *Uapaca vanhoutei* \| Eurphorbiaceae \| 0 \| 2 \| 0.613 \| medium \| small \| biotic \| non-pioneer \| narrow \|  \| \|  \| *Vitex cienkowskii* \| Verbenaceae \| 0 \| 9 \| 0.532 \|  \|  \|  \|  \|  \|  \| \|  \| *Vitex grandifolia* \| Verbenaceae \| 0 \| 1 \| 0.532 \| medium \| medium \| biotic \| non-pioneer \| narrow \|  \| \|  \| *Xylopia aethiopica* \| Annonaceae \| 0 \| 2 \| 0.475 \| large \| medium \| biotic \| non-pioneer \| narrow \|  \| \|  \| *Xylopia hypolampra* \| Annonaceae \| 1 \| 3 \| 0.643 \| medium \| medium \| biotic \| non-pioneer \| narrow \|  \| \|  \| *Xylopia paviflora* \| Annonaceae \| 1 \| 0 \| 0.810 \| medium \| large \| biotic \| non-pioneer \| narrow \|  \| \|  \| *Xylopia quintasii* \| Annonaceae \| 1 \| 14 \| 0.804 \| medium \| medium \| biotic \| non-pioneer \| narrow \|  \| \|  \| *Xylopia* sp. A \| Annonaceae \| 0 \| 1 \| 0.653 \|  \|  \|  \|  \|  \|  \| \|  \| *Xylopia* sp. B \| Annonaceae \| 0 \| 1 \| 0.653 \|  \|  \|  \|  \|  \|  \| \|  \| *Xylopia staudtii* \| Annonaceae \| 0 \| 1 \| 0.439 \| medium \|  \|  \| non-pioneer \|  \|  \| \|  \| *Zanthoxylum giletii* \| Rutaceae \| 0 \| 2 \| 0.705 \| medium \| large \| biotic \| non-pioneer \| narrow \|  \| \|  \| *Zanthoxylum heitzii* \| Rutaceae \| 0 \| 1 \| 0.451 \| medium \| large \| biotic \| non-pioneer \| narrow \|  \| |
| --- | --- | --- | --- | --- | --- | --- | --- | --- | --- | --- | --- | --- | --- | --- | --- | --- | --- | --- | --- | --- | --- | --- | --- | --- | --- | --- | --- | --- | --- | --- | --- | --- | --- | --- | --- | --- | --- | --- | --- | --- | --- | --- | --- | --- | --- | --- | --- | --- | --- | --- | --- | --- | --- | --- | --- | --- | --- | --- | --- | --- | --- | --- | --- | --- | --- | --- | --- | --- | --- | --- | --- | --- | --- | --- | --- | --- | --- | --- | --- | --- | --- | --- | --- | --- | --- | --- | --- | --- | --- | --- | --- | --- | --- | --- | --- | --- | --- | --- | --- | --- | --- | --- | --- | --- | --- | --- | --- | --- | --- | --- | --- | --- | --- | --- | --- | --- | --- | --- | --- | --- | --- | --- | --- | --- | --- | --- | --- | --- | --- | --- | --- | --- | --- | --- | --- | --- | --- | --- | --- | --- | --- | --- | --- | --- | --- | --- | --- | --- | --- | --- | --- | --- | --- | --- | --- | --- | --- | --- | --- | --- | --- | --- | --- | --- | --- | --- | --- | --- | --- | --- | --- | --- | --- | --- | --- | --- | --- | --- | --- | --- | --- | --- | --- | --- | --- | --- | --- | --- | --- | --- | --- | --- | --- | --- | --- | --- | --- | --- | --- | --- | --- | --- | --- | --- | --- | --- | --- | --- | --- | --- | --- | --- | --- | --- | --- | --- | --- | --- | --- | --- | --- | --- | --- | --- | --- | --- | --- | --- | --- | --- | --- | --- | --- | --- | --- | --- | --- | --- | --- | --- | --- | --- | --- | --- | --- | --- | --- | --- | --- | --- | --- | --- | --- | --- | --- | --- | --- | --- | --- | --- | --- | --- | --- | --- | --- | --- | --- | --- | --- | --- | --- | --- | --- | --- | --- | --- | --- | --- | --- | --- | --- | --- | --- | --- | --- | --- | --- | --- | --- | --- | --- | --- | --- | --- | --- | --- | --- | --- | --- | --- | --- | --- | --- | --- | --- | --- | --- | --- | --- | --- | --- | --- | --- | --- | --- | --- | --- | --- | --- | --- | --- | --- | --- | --- | --- | --- | --- | --- | --- | --- | --- | --- | --- | --- | --- | --- | --- | --- | --- | --- | --- | --- | --- | --- | --- | --- | --- | --- | --- | --- | --- | --- | --- | --- | --- | --- | --- | --- | --- | --- | --- | --- | --- | --- | --- | --- | --- | --- | --- | --- | --- | --- | --- | --- | --- | --- | --- | --- | --- | --- | --- | --- | --- | --- | --- | --- | --- | --- | --- | --- | --- | --- | --- | --- | --- | --- | --- | --- | --- | --- | --- | --- | --- | --- | --- | --- | --- | --- | --- | --- | --- | --- | --- | --- | --- | --- | --- | --- | --- | --- | --- | --- | --- | --- | --- | --- | --- | --- | --- | --- | --- | --- | --- | --- | --- | --- | --- | --- | --- | --- | --- | --- | --- | --- | --- | --- | --- | --- | --- | --- | --- | --- | --- | --- | --- | --- | --- | --- | --- | --- | --- | --- | --- | --- | --- | --- | --- | --- | --- | --- | --- | --- | --- | --- | --- | --- | --- | --- | --- | --- | --- | --- | --- | --- | --- | --- | --- | --- | --- | --- | --- | --- | --- | --- | --- | --- | --- | --- | --- | --- | --- | --- | --- | --- | --- | --- | --- | --- | --- | --- | --- | --- | --- | --- | --- | --- | --- | --- | --- | --- | --- | --- | --- | --- | --- | --- | --- | --- | --- | --- | --- | --- | --- | --- | --- | --- | --- | --- | --- | --- | --- | --- | --- | --- | --- | --- | --- | --- | --- | --- | --- | --- | --- | --- | --- | --- | --- | --- | --- | --- | --- | --- | --- | --- | --- | --- | --- | --- | --- | --- | --- | --- | --- | --- | --- | --- | --- | --- | --- | --- | --- | --- | --- | --- | --- | --- | --- | --- | --- | --- | --- | --- | --- | --- | --- | --- | --- | --- | --- | --- | --- | --- | --- | --- | --- | --- | --- | --- | --- | --- | --- | --- | --- | --- | --- | --- | --- | --- | --- | --- | --- | --- | --- | --- | --- | --- | --- | --- | --- | --- | --- | --- | --- | --- | --- | --- | --- | --- | --- | --- | --- | --- | --- | --- | --- | --- | --- | --- | --- | --- | --- | --- | --- | --- | --- | --- | --- | --- | --- | --- | --- | --- | --- | --- | --- | --- | --- | --- | --- | --- | --- | --- | --- | --- | --- | --- | --- | --- | --- | --- | --- | --- | --- | --- | --- | --- | --- | --- | --- | --- | --- | --- | --- | --- | --- | --- | --- | --- | --- | --- | --- | --- | --- | --- | --- | --- | --- | --- | --- | --- | --- | --- | --- | --- | --- | --- | --- | --- | --- | --- | --- | --- | --- | --- | --- | --- | --- | --- | --- | --- | --- | --- | --- | --- | --- | --- | --- | --- | --- | --- | --- | --- | --- | --- | --- | --- | --- | --- | --- | --- | --- | --- | --- | --- | --- | --- | --- | --- | --- | --- | --- | --- | --- | --- | --- | --- | --- | --- | --- | --- | --- | --- | --- | --- | --- | --- | --- | --- | --- | --- | --- | --- | --- | --- | --- | --- | --- | --- | --- | --- | --- | --- | --- | --- | --- | --- | --- | --- | --- | --- | --- | --- | --- | --- | --- | --- | --- | --- | --- | --- | --- | --- | --- | --- | --- | --- | --- | --- | --- | --- | --- | --- | --- | --- | --- | --- | --- | --- | --- | --- | --- | --- | --- | --- | --- | --- | --- | --- | --- | --- | --- | --- | --- | --- | --- | --- | --- | --- | --- | --- | --- | --- | --- | --- | --- | --- | --- | --- | --- | --- | --- | --- | --- | --- | --- | --- | --- | --- | --- | --- | --- | --- | --- | --- | --- | --- | --- | --- | --- | --- | --- | --- | --- | --- | --- | --- | --- | --- | --- | --- | --- | --- | --- | --- | --- | --- | --- | --- | --- | --- | --- | --- | --- | --- | --- | --- | --- | --- | --- | --- | --- | --- | --- | --- | --- | --- | --- | --- | --- | --- | --- | --- | --- | --- | --- | --- | --- | --- | --- | --- | --- | --- | --- | --- | --- | --- | --- | --- | --- | --- | --- | --- | --- | --- | --- | --- | --- | --- | --- | --- | --- | --- | --- | --- | --- | --- | --- | --- | --- | --- | --- | --- | --- | --- | --- | --- | --- | --- | --- | --- | --- | --- | --- | --- | --- | --- | --- | --- | --- | --- | --- | --- | --- | --- | --- | --- | --- | --- | --- | --- | --- | --- | --- | --- | --- | --- | --- | --- | --- | --- | --- | --- | --- | --- | --- | --- | --- | --- | --- | --- | --- | --- | --- | --- | --- | --- | --- | --- | --- | --- | --- | --- | --- | --- | --- | --- | --- | --- | --- | --- | --- | --- | --- | --- | --- | --- | --- | --- | --- | --- | --- | --- | --- | --- | --- | --- | --- | --- | --- | --- | --- | --- | --- | --- | --- | --- | --- | --- | --- | --- | --- | --- | --- | --- | --- | --- | --- | --- | --- | --- | --- | --- | --- | --- | --- | --- | --- | --- | --- | --- | --- | --- | --- | --- | --- | --- | --- | --- | --- | --- | --- | --- | --- | --- | --- | --- | --- | --- | --- | --- | --- | --- | --- | --- | --- | --- | --- | --- | --- | --- | --- | --- | --- | --- | --- | --- | --- | --- | --- | --- | --- | --- | --- | --- | --- | --- | --- | --- | --- | --- | --- | --- | --- | --- | --- | --- | --- | --- | --- | --- | --- | --- | --- | --- | --- | --- | --- | --- | --- | --- | --- | --- | --- | --- | --- | --- | --- | --- | --- | --- | --- | --- | --- | --- | --- | --- | --- | --- | --- | --- | --- | --- | --- | --- | --- | --- | --- | --- | --- | --- | --- | --- | --- | --- | --- | --- | --- | --- | --- | --- | --- | --- | --- | --- | --- | --- | --- | --- | --- | --- | --- | --- | --- | --- | --- | --- | --- | --- | --- | --- | --- | --- | --- | --- | --- | --- | --- | --- | --- | --- | --- | --- | --- | --- | --- | --- | --- | --- | --- | --- | --- | --- | --- | --- | --- | --- | --- | --- | --- | --- | --- | --- | --- | --- | --- | --- | --- | --- | --- | --- | --- | --- | --- | --- | --- | --- | --- | --- | --- | --- | --- | --- | --- | --- | --- | --- | --- | --- | --- | --- | --- | --- | --- | --- | --- | --- | --- | --- | --- | --- | --- | --- | --- | --- | --- | --- | --- | --- | --- | --- | --- | --- | --- | --- | --- | --- | --- | --- | --- | --- | --- | --- | --- | --- | --- | --- | --- | --- | --- | --- | --- | --- | --- | --- | --- | --- | --- | --- | --- | --- | --- | --- | --- | --- | --- | --- | --- | --- | --- | --- | --- | --- | --- | --- | --- | --- | --- | --- | --- | --- | --- | --- | --- | --- | --- | --- | --- | --- | --- | --- | --- | --- | --- | --- | --- | --- | --- | --- | --- | --- | --- | --- | --- | --- | --- | --- | --- | --- | --- | --- | --- | --- | --- | --- | --- | --- | --- | --- | --- | --- | --- | --- | --- | --- | --- | --- | --- | --- | --- | --- | --- | --- | --- | --- | --- | --- | --- | --- | --- | --- | --- | --- | --- | --- | --- | --- | --- | --- | --- | --- | --- | --- | --- | --- | --- | --- | --- | --- | --- | --- | --- | --- | --- | --- | --- | --- | --- | --- | --- | --- | --- | --- | --- | --- | --- | --- | --- | --- | --- | --- | --- | --- | --- | --- | --- | --- | --- | --- | --- | --- | --- | --- | --- | --- | --- | --- | --- | --- | --- | --- | --- | --- | --- | --- | --- | --- | --- | --- | --- | --- | --- | --- | --- | --- | --- | --- | --- | --- | --- | --- | --- | --- | --- | --- | --- | --- | --- | --- | --- | --- | --- | --- | --- | --- | --- | --- | --- | --- | --- | --- | --- | --- | --- | --- | --- | --- | --- | --- | --- | --- | --- | --- | --- | --- | --- | --- | --- | --- | --- | --- | --- | --- | --- | --- | --- | --- | --- | --- | --- | --- | --- | --- | --- | --- | --- | --- | --- | --- | --- | --- | --- | --- | --- | --- | --- | --- | --- | --- | --- | --- | --- | --- | --- | --- | --- | --- | --- | --- | --- | --- | --- | --- | --- | --- | --- | --- | --- | --- | --- | --- | --- | --- | --- | --- | --- | --- | --- | --- | --- | --- | --- | --- | --- | --- | --- | --- | --- | --- | --- | --- | --- | --- | --- | --- | --- | --- | --- | --- | --- | --- | --- | --- | --- | --- | --- | --- | --- | --- | --- | --- | --- | --- | --- | --- | --- | --- | --- | --- | --- | --- | --- | --- | --- | --- | --- | --- | --- | --- | --- | --- | --- | --- | --- | --- | --- | --- | --- | --- | --- | --- | --- | --- | --- | --- | --- | --- | --- | --- | --- | --- | --- | --- | --- | --- | --- | --- | --- | --- | --- | --- | --- | --- | --- | --- | --- | --- | --- | --- | --- | --- | --- | --- | --- | --- | --- | --- | --- | --- | --- | --- | --- | --- | --- | --- | --- | --- | --- | --- | --- | --- | --- | --- | --- | --- | --- | --- | --- | --- | --- | --- | --- | --- | --- | --- | --- | --- | --- | --- | --- | --- | --- | --- | --- | --- | --- | --- | --- | --- | --- | --- | --- | --- | --- | --- | --- | --- | --- | --- | --- | --- | --- | --- | --- | --- | --- | --- | --- | --- | --- | --- | --- | --- | --- | --- | --- | --- | --- | --- | --- | --- | --- | --- | --- | --- | --- | --- | --- | --- | --- | --- | --- | --- | --- | --- | --- | --- | --- | --- | --- | --- | --- | --- | --- | --- | --- | --- | --- | --- | --- | --- | --- | --- | --- | --- | --- | --- | --- | --- | --- | --- | --- | --- | --- | --- | --- | --- | --- | --- | --- | --- | --- | --- | --- | --- | --- | --- | --- | --- | --- | --- | --- | --- | --- | --- | --- | --- | --- | --- | --- | --- | --- | --- | --- | --- | --- | --- | --- | --- | --- | --- | --- | --- | --- | --- | --- | --- | --- | --- | --- | --- | --- | --- | --- | --- | --- | --- | --- | --- | --- | --- | --- | --- | --- | --- | --- | --- | --- | --- | --- | --- | --- | --- | --- | --- | --- | --- | --- | --- | --- | --- | --- | --- | --- | --- | --- | --- | --- | --- | --- | --- | --- | --- | --- | --- | --- | --- | --- | --- | --- | --- | --- | --- | --- | --- | --- | --- | --- | --- | --- | --- | --- | --- | --- | --- | --- | --- | --- | --- | --- | --- | --- | --- | --- | --- | --- | --- | --- | --- | --- | --- | --- | --- | --- | --- | --- | --- | --- | --- | --- | --- | --- | --- | --- | --- | --- | --- | --- | --- | --- | --- | --- | --- | --- | --- | --- | --- | --- | --- | --- | --- | --- | --- | --- | --- | --- | --- | --- | --- | --- | --- | --- | --- | --- | --- | --- | --- | --- | --- | --- | --- | --- | --- | --- | --- | --- | --- | --- | --- | --- | --- | --- | --- | --- | --- | --- | --- | --- | --- | --- | --- | --- | --- | --- | --- | --- | --- | --- | --- | --- | --- | --- | --- | --- | --- | --- | --- | --- | --- | --- | --- | --- | --- | --- | --- | --- | --- | --- | --- | --- | --- | --- | --- | --- | --- | --- | --- | --- | --- | --- | --- | --- | --- | --- | --- | --- | --- | --- | --- | --- | --- | --- | --- | --- | --- | --- | --- | --- | --- | --- | --- | --- | --- | --- | --- | --- | --- | --- | --- | --- | --- | --- | --- | --- | --- | --- | --- | --- | --- | --- | --- | --- | --- | --- | --- | --- | --- | --- | --- | --- | --- | --- | --- | --- | --- | --- | --- | --- | --- | --- | --- | --- | --- | --- | --- | --- | --- | --- | --- | --- | --- | --- | --- | --- | --- | --- | --- | --- | --- | --- | --- | --- | --- | --- | --- | --- | --- | --- | --- | --- | --- | --- | --- | --- | --- | --- | --- | --- | --- | --- | --- | --- | --- | --- | --- | --- | --- | --- | --- | --- | --- | --- | --- | --- | --- | --- | --- | --- | --- | --- | --- | --- | --- | --- | --- | --- | --- | --- | --- | --- | --- | --- | --- | --- | --- | --- | --- | --- | --- | --- | --- | --- | --- | --- | --- | --- | --- | --- | --- | --- | --- | --- | --- | --- | --- | --- | --- | --- | --- | --- | --- | --- | --- | --- | --- | --- | --- | --- | --- | --- | --- | --- | --- | --- | --- | --- | --- | --- | --- | --- | --- | --- | --- | --- | --- | --- | --- | --- | --- | --- | --- | --- | --- | --- | --- | --- | --- | --- | --- | --- | --- | --- | --- | --- | --- | --- | --- | --- | --- | --- | --- | --- | --- | --- | --- | --- | --- | --- | --- | --- | --- | --- | --- | --- | --- | --- | --- | --- | --- | --- | --- | --- | --- | --- | --- | --- | --- | --- | --- | --- | --- | --- | --- | --- | --- | --- | --- | --- | --- | --- | --- | --- | --- | --- | --- | --- | --- | --- | --- | --- | --- | --- | --- | --- | --- | --- | --- | --- | --- | --- | --- | --- | --- | --- | --- | --- | --- | --- | --- | --- | --- | --- | --- | --- | --- | --- | --- | --- | --- | --- | --- | --- | --- | --- | --- | --- | --- | --- | --- | --- | --- | --- | --- | --- | --- | --- | --- | --- | --- | --- | --- | --- | --- | --- | --- | --- | --- | --- | --- | --- | --- | --- | --- |
